# Shape Memory Polymer Composites: 4D Printing, Smart Structures, and Applications

**DOI:** 10.34133/research.0234

**Published:** 2023-11-07

**Authors:** Shiyu Yan, Fenghua Zhang, Lan Luo, Linlin Wang, Yanju Liu, Jinsong Leng

**Affiliations:** ^1^ Centre for Composite Materials and Structures, Harbin Institute of Technology (HIT), No.2 Yikuang Street, Harbin 150000, People’s Republic of China.; ^2^ Department of Astronautic Science and Mechanics, Harbin Institute of Technology (HIT), No. 92 West Dazhi Street, Harbin 150000, People’s Republic of China.

## Abstract

Shape memory polymers (SMPs) and their composites (SMPCs) are smart materials that can be stably deformed and then return to their original shape under external stimulation, thus having a memory of their shape. Three-dimensional (3D) printing is an advanced technology for fabricating products using a digital software tool. Four-dimensional (4D) printing is a new generation of additive manufacturing technology that combines shape memory materials and 3D printing technology. Currently, 4D-printed SMPs and SMPCs are gaining considerable research attention and are finding use in various fields, including biomedical science. This review introduces SMPs, SMPCs, and 4D printing technologies, highlighting several special 4D-printed structures. It summarizes the recent research progress of 4D-printed SMPs and SMPCs in various fields, with particular emphasis on biomedical applications. Additionally, it presents an overview of the challenges and development prospects of 4D-printed SMPs and SMPCs and provides a preliminary discussion and useful reference for the research and application of 4D-printed SMPs and SMPCs.

## Introduction

Shape memory polymers (SMPs) are an important category of smart materials that can change their original shape to any desired temporary shape under the coordinated action of external forces and stimuli. SMPs can then recover their initial shape when stimulated again, completing a shape memory cycle [1]. Moreover, a variety of functional fillers are introduced into the SMPs to prepare shape memory polymer composites (SMPCs). This gives SMPs a wider range of stimulus forms, such as temperature, current, light, magnetism, or chemical solutions. SMPs and SMPCs exhibit different shape memory effects (SMEs); these can include the memory of multiple shapes and reversible deformations [2]. Since 1984, when the French company Cdf Chime developed the first SMP, polynorbornene, SMPs have attracted considerable attention owing to their low weight and cost, good molding processability, large deformation, strong shape recovery ability, mild shape recovery conditions, and other valuable characteristics [3–6]. As more SMPs and SMPCs with excellent properties are introduced, their potential applications in various fields are beginning to be explored. Currently, SMPs and SMPCs are mainly used in aerospace [7], additive manufacturing (AM) [2], electronic packaging [8], and smart textiles [9]. Furthermore, their biomedical applications are particularly outstanding [10,11]. For example, SMPs and SMPCs have been used in many biomedical tools, such as grippers, surgical sutures, aneurysm occluders, tracheal stents, and vascular stents [12,13].

Three-dimensional (3D) printing technology, also known as AM, is an advanced technology that predefines complex structures with the help of digital software programs to prepare the desired product without requiring molds. Since Chuck Hull founded 3D Systems in 1986, 3D printing technology has attracted wide attention. With the gradual development of materials, printers, and processes, 3D printing technology has been applied to fields as diverse as aerospace, biomedical, and food processing [14–21]. Compared to traditional manufacturing, 3D printing technology streamlines the production process and shortens production time. It realizes multimaterial printing and provides unparalleled flexibility and convenience [22–24].

In February 2013, Tibbits from the Massachusetts Institute of Technology introduced the concept of 4-dimensional (4D) printing technology at a TED conference, defining it as a new design of complex structures that can change themselves over time in response to external stimuli [25]. “Programmable” is a important feature that distinguishes 4D printing from 3D printing. 4D printing can prepare geometrically complex and highly personalized structures on demand. The core of 4D printing technology is how to achieve programmable shape changes through the design of materials, structures, and external excitation conditions. Among them, smart materials are the foundation of 4D printing. The structure obtained by using smart material 4D printing can undergo self-transformation of its physical properties and functions over time under specific environmental stimuli. There are many smart materials that can be used for 4D printing, such as shape memory alloys, shape memory ceramics, SMPs, SMPCs, etc. Compared to other smart materials, SMPs and SMPCs have the advantages of low density, low cost, variety, and designability, so 4D printing related to them is most favored by researchers [26–30].

**Fig. 1.**
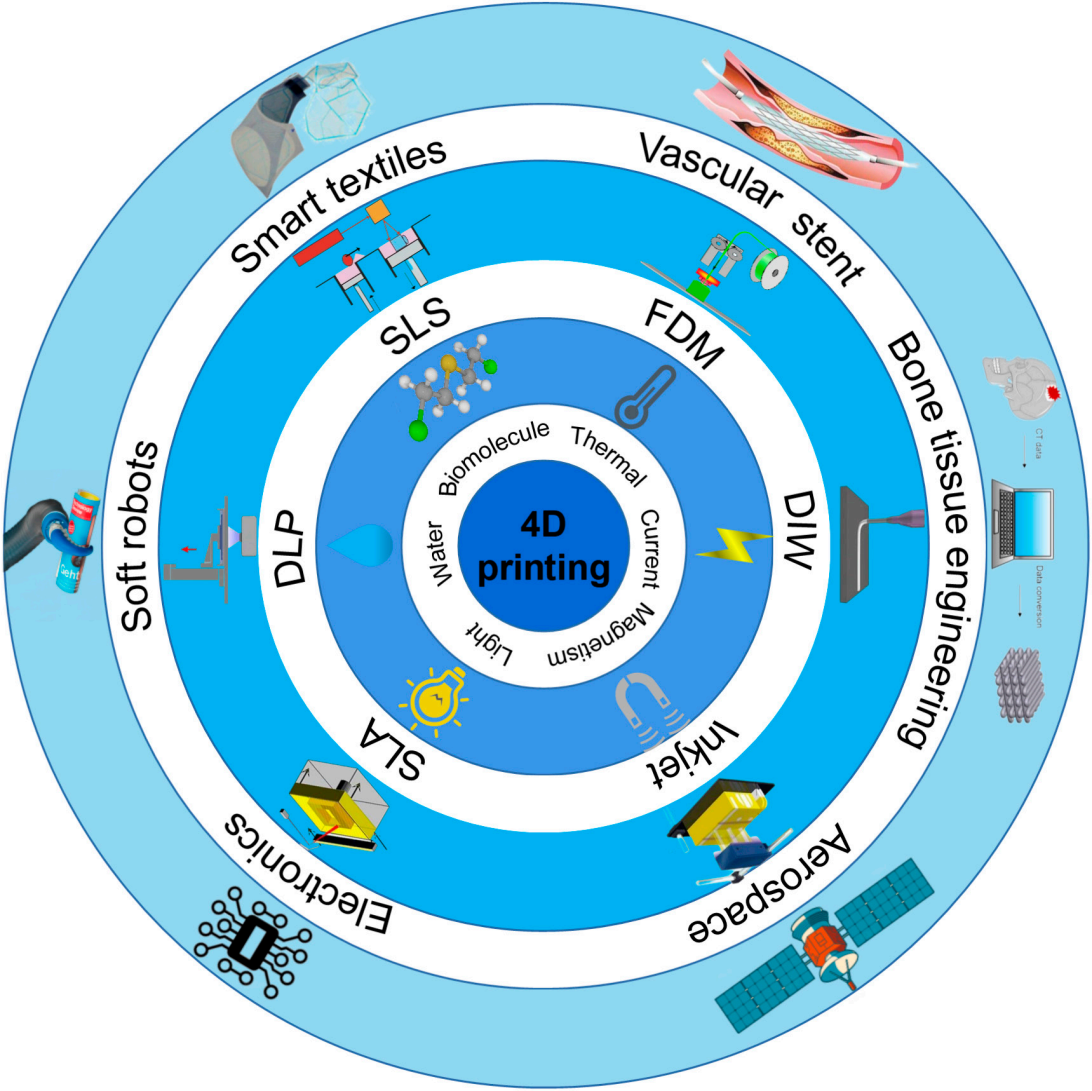
Schematic of the development of 4D printing: actuation methods, printing technology, and applications in the various fields.

With addition of time as the fourth dimension, the 4D printing technology allows the fast development of “living” adaptable structures that enables the growth opportunities in various fields. Ge et al. [31] developed an active hinge through 4D printing and used it to prepare a morphing wing flap and a deployable structure, demonstrating that 4D printing has great practical value. Today, 4D printing processes include fused deposition modeling (FDM), inkjet printing, direct ink writing (DIW), stereolithography apparatus (SLA), digital light processing (DLP), selective laser sintering (SLS), and more [32–36]. As the 4D printing technology is studied further, its advantages have become evident. Compared with 3D printing, 4D printing can not only perform shape changes but also realize a variety of amazing functions such as self-transformation, self-assembly, and self-healing through predefined deformation schemes (including the shape, properties, and functions of the components) while maintaining its excellent quality, precision, and performance [37–44]. Therefore, 4D printing technology has great potential for applications in various fields, such as construction, agriculture, soft robots, electronics, smart furniture, aerospace, textile materials, and biomedical devices (Fig. 1) [14,31,45–52].

## Shape Memory Mechanism

The continual in-depth study of SMPs has gradually expanded the understanding of their shape memory mechanism. SME is related to the molecular structure of SMPs, which has 2 essential components: the cross-linked network and the reversible unit. Specifically, the reversible unit generally refers to the component of the polymer that can undergo phase transitions, such as glass transition, melting and crystallization, etc. The reversible unit is used to control the macroscopic shape of the polymer, whereas the cross-linked network provides the driving force for shape recovery as well as imparts its initial shape [53–56]. When the cross-linked network is physically cross-linked, SMPs are thermoplastic; thermoplastic SMPs include polylactic acid (PLA), polycaprolactone (PCL), thermoplastic polyurethane (PU), etc. When chemically cross-linked, SMPs are thermosetting; thermosetting SMPs include epoxy resin, cyanate resin, thermosetting PU, polyimide, polystyrene, etc.

**Fig. 2.**
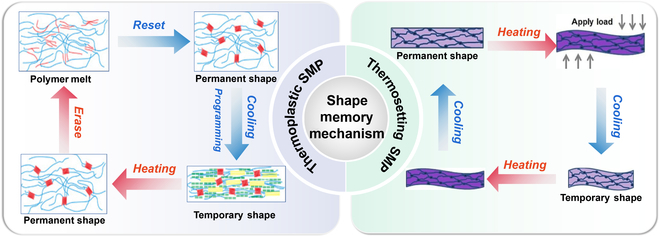
Schematic of shape memory mechanism. All images reproduced with permission: “Thermoplastic SMP” from [59]. Copyright 2017 American Chemical Society. “Thermosetting SMP” from [61]. Copyright 2019 Wiley-VCH.

Both thermosetting and thermoplastic SMPs have the same structural molecular changes during their shape memory process, as shown in Fig. 2. The long lines in the figure represent the reversible unit, and the nodes correspond to the cross-linked network. At temperatures below the transition temperature of the SMP (*T*_*t*_), the reversible unit and cross-linked network are in their frozen states, and the SMP maintains its permanent shape. When the temperature rises above *T*_*t*_, the chain segment movement of the reversible unit intensifies, making it highly flexible and able to undergo arbitrary deformation by the action of an external force. In the predeformation process, the cross-linked network remains frozen, while the chain segments of the reversible unit are changed from a twisted state to an ordered state by the external force. When the temperature is reduced to below *T*_*t*_, the reversible unit freezes, and the temporary shape of the SMP is fixed. However, when the temperature rises above *T*_*t*_ again, the reversible unit is no longer frozen, and its chain segments will return to the initial state under the action of internal stress and molecular thermal motion, resulting in the SMP returning to its permanent shape [57,58].

However, there are some differences in the properties of thermoplastic and thermosetting SMPs due to the different forms of cross-linking between them. Chemical cross-linking leads to thermoset SMPs with strong shape memory, but at the cost of reduced reworkability, and their permanent shape cannot be redefined. Thermoplastic SMPs can redefine their permanent shape, but their shape memory performance is also reduced compared to thermosetting SMPs [59–61]. In addition, they are suitable for different printing technologies. Thermosetting SMPs are often used in DIW, SLA, and DLP, while thermoplastic SMPs are mainly used in FDM.

## 4D Printing Technology

As AM technology has developed, the number of processes available for 4D printing has gradually increased, which has promoted the research and application of SMPs and SMPCs in various fields.

### Fused deposition modeling

**Fig. 3.**
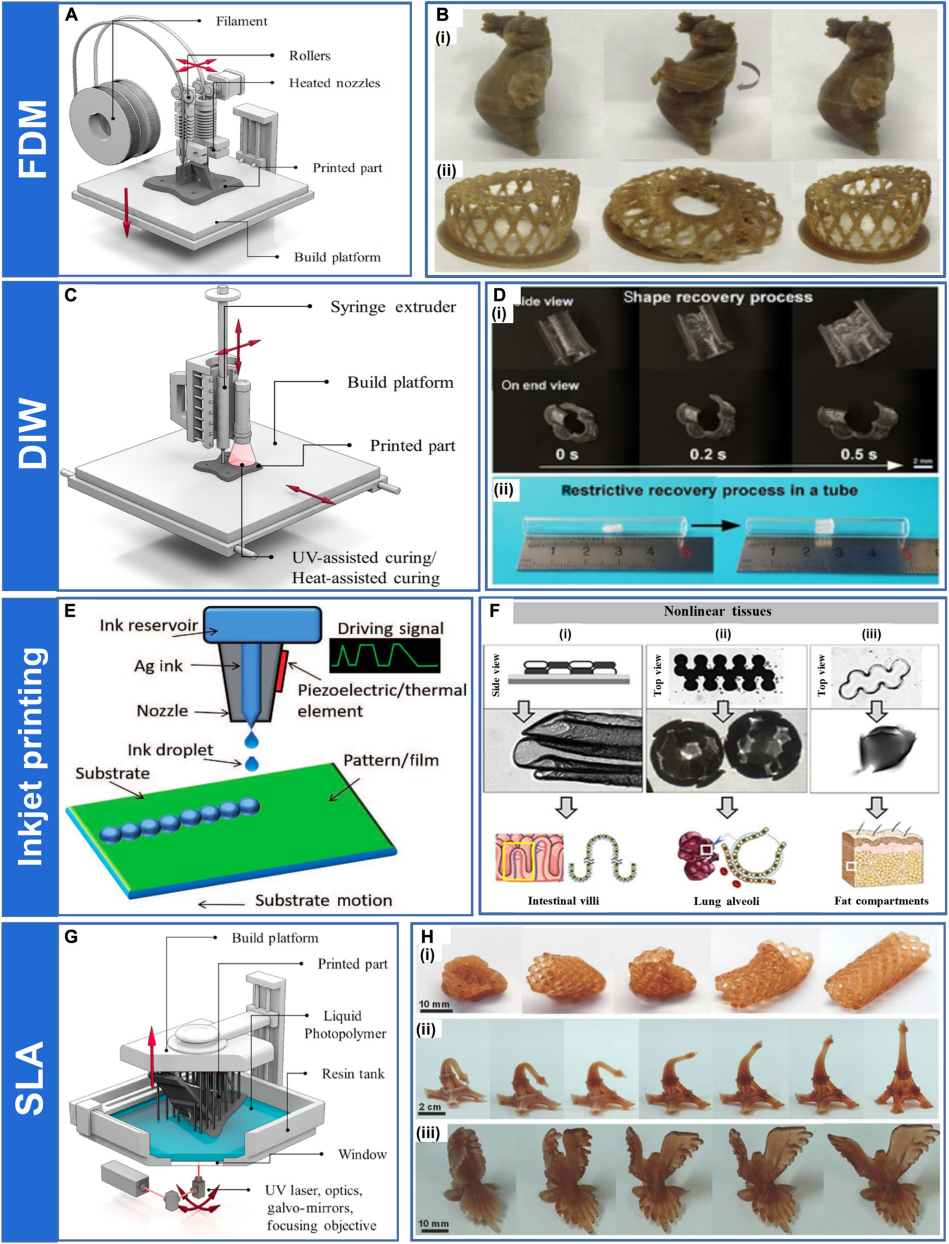
(A) Schematic of the printing process of FDM. Reproduced with permission from [79]. Copyright 2020 Elsevier. (B) Demonstration of some 4D-printed models: (i) Kungfu Panda and (ii) “Bird's Nest”. Reproduced with permission from [67]. Copyright 2020 Elsevier. (C) Schematic of the printing process of DIW. Reproduced with permission from [79]. Copyright 2020 Elsevier. (D) (i) Macroscopic shape memory behavior of the 4D-printed stent within 0.5 s and (ii) shape recovery process in the tube. Reproduced with permission from [73]. Copyright 2019 Wiley-VCH. (E) Schematic of the printing process of inkjet printing. Reproduced with permission from [74]. Copyright 2019 IntechOpen. (F) Inkjet-printing biomaterial inks by different layouts and patterns resulted in various kinds of self-folded 3D microstructures: (i) groove and ridge-like scaffold, (ii) porous hollow sphere, and (iii) hollow sphere. Reproduced with permission from [78]. Copyright 2020 IOP Publishing. (G) Schematic of the printing process of SLA. Reproduced with permission from [79]. Copyright 2020 Elsevier. (H) Complex structures printed using SLA: (i) a model cardiovascular stent, (ii) an Eiffel Tower model, and (iii) a bird. Reproduced with permission from [80]. Copyright 2016 Wiley-VCH.

FDM is a low-cost, easy-to-use 4D printing technology that does not require chemical reactions during the printing process [62]. In the process of using FDM, thermoplastic solid filaments are heated and melted in the nozzle, which is computer-controlled to move along a prescribed route. As the nozzle moves, the melted material is extruded to form 3D objects on the base in a line-by-line, layer-by-layer fashion (Fig. 3A) [63]. The materials commonly used in FDM include PLA, PCL, polycarbonate, polyethylene terephthalate, and polyphenyl sulfoxide.

Popescu et al. [64] investigated the influence of the process parameters of FDM on the mechanical properties of the fabricated polymeric parts. The most marked parameters included slicing parameters, molding direction, temperature conditions, and filament bonding. Tian et al. [65] proposed a novel FDM process whereby they prepared PLA and continuous carbon fibers into continuous fiber-reinforced thermoplastic composites that exhibited improved properties. Wang et al. [66] utilized 4D printing technology parameters to achieve multishape memory and variable recovery force editing of materials. Cheng et al. [67] developed an ultraviolet (UV)-assisted FDM 4D printing method and used it to prepare 4D-printed models to evaluate their printability and SME (Fig. 3B). The 4D printing method is expected to be used to prepare medical protective devices for personalized and patient-adaptive treatments. Lin et al. [68] developed a shape memory composite with polybutylene succinate and PLA as a novel wire for FDM 4D printing, and its embolization process in an aneurysm model demonstrated its promising future in the biomedical field.

### Direct ink writing

DIW is an AM method that extrudes viscoelastic ink through a nozzle at high pressure and moves in a computer-controlled dispenser following a predetermined trajectory to build the geometry layer-by-layer (Fig. 3C). Viscoelastic inks for DIW require both shear thinning behavior and rapid pseudoplastic-to-dilatant recovery to ensure the smooth extrusion of the ink from the nozzle and shape retention after ink deposition, respectively. Because the rheological properties of the ink will affect the effectiveness of DIW printing, the ink viscosity adjustment is crucial. Many methods are used to adjust ink viscosity, such as using solvents, high- or low-temperature printing, adding rheological modifiers, and UV assistance [69].

Shi et al. [70] developed a new method for preparing microcrystalline glass ink that enabled the recycling of thermosetting polymers for DIW printing. Chen et al. [71] proposed a novel printing technique. They used UV light to help materials cure during DIW printing, which can be used to prepare thermoset materials with high tensile toughness. Wei et al. [72] used DIW to print UV cross-linked PLA-based 3D structures with Fe_3_O_4_ added to the system to enable the remote actuation of the object by magnetic field action, which has great potential for application in minimally invasive medical procedures. Wan et al. [73] prepared a 4D-printed stent based on poly(d,l-lactide-co-trimethylene carbonate) by DIW. They demonstrated that it could perform rapid shape recovery near human body temperature with excellent shape recovery (Fig. 3D).

### Inkjet printing

Inkjet printing is one of the most popular 4D printing technologies. In inkjet printing, multiple nozzles of the device can work simultaneously to eject different liquid resins on the printing platform, and the stacked ink is cured to form the desired geometric components (Fig. 3E) [14,74].

Ge et al. [75] were the first to use inkjet printing technology for 4D printing and successfully produced deformable composites. Xu et al. [76] applied inkjet printing to the biomedical field and proposed a 3D inkjet bioprinting system. The bioprinting system has successfully fabricated fibroblast (3T3 cells) base tubes with an overhang structure; the survival rate of the printed 3T3 cells exceeded 82% after a 72-h culture period. Mao et al. [77] used inkjet printing to produce a new “active material” type that could achieve reversible deformation behavior without external forces. Cui et al. [78] created a 4D inkjet printing platform and used it to produce cell-encapsulated hollow 3D stents. This solves some of the problems faced by 4D printing for tissue engineering applications (Fig. 3F).

### Stereolithography apparatus

SLA was introduced by Charles Hull in 1986, and it is one of the first printing methods implemented in industry and one of the most widely used AM technologies today. SLA uses liquid photocuring polymers as raw materials, which are loaded into resin storage tanks. High-power lasers are fired from below the resin storage tanks through transparent windows, solidifying the liquid materials on the surface of printed components. As the printing platform moves up and the laser source moves, the laser prints out the desired geometry layer-by-layer in a sequence from point to line and then from line to surface (Fig. 3G) [79].

Choong et al. [33] investigated the relationship between energy density and curing depth in SLA, finding that the curing depth of the material increases with cross-linker content, but the shape memory properties decrease. Zarek et al. [80] successfully prepared a variety of complex 4D structures using SLA and verified the shape memory properties of the printed objects under thermal stimulation (Fig. 3H). Lu et al. [81] developed a magnetic field-assisted SLA in which magnetic fillers are controllably distributed in liquid resin with the help of an external magnetic field and can be distributed in a specific pattern. This can be used to prepare various novel smart structures, opening the way for applying 4D-printed magnetically driven SMPCs to the biomedical field.

### Digital light processing

**Fig. 4.**
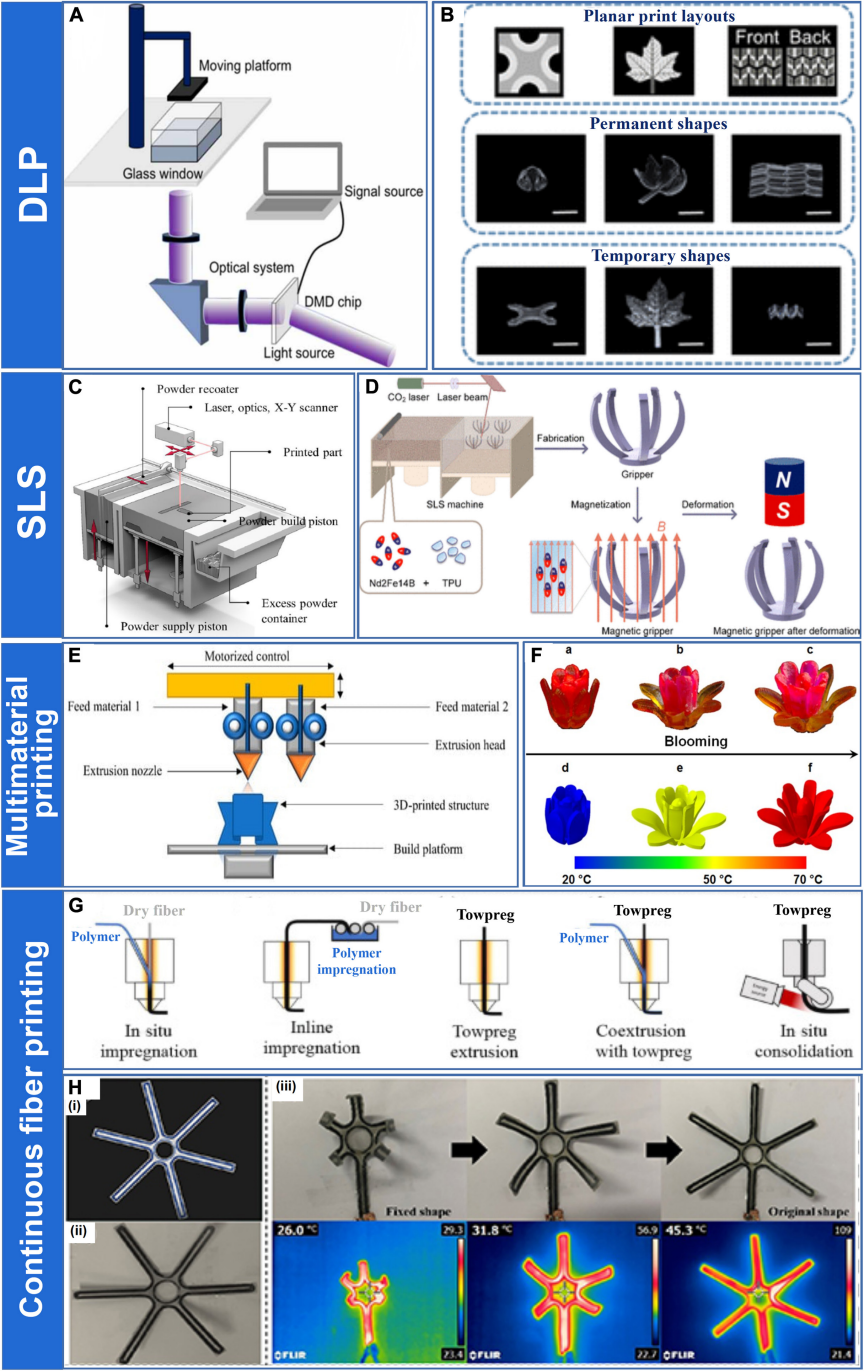
(A) Schematic of the printing process of DLP. Reproduced with permission from [82].Copyright 2022 IOP Publishing. (B) Digital fabrication of complex 4D-printed structures. Reproduced with permission from [83]. Copyright 2019 American Chemical Society. (C) Schematic of the printing process of SLS. Reproduced with permission from [79]. Copyright 2020 Elsevier. (D) Schematic diagram of the SLS fabrication and magnetization process of the magnetism-responsive gripper. Reproduced with permission from [93]. Copyright 2021 American Chemical Society. (E) Schematic of the printing process of multimaterial printing. Reproduced with permission from [97]. Copyright 2022 Multidisciplinary Digital Publishing Institute. (F) The sequential recovery of a multimaterial flower. Reproduced with permission from [47]. Copyright 2016 Springer Nature. (G) Schematic of the printing process of continuous fiber printing. Reproduced with permission from [102]. Copyright 2023 Elsevier. (H) The continuous carbon fiber reinforced SMPC as a deployable claw device: (i) model, (ii) printed specimen, and (iii) the snapshots and infrared images of the claw device during the electrothermal-actuated shape recovery process. Reproduced with permission from [106]. Copyright 2021 IOP Publishing.

The technological progress of DLP is mainly driven by the development of the digital micromirror device chip developed by J. Hornbeck in 1987. Compared with traditional SLA, DLP differs only in the curing method, which uses a digital light projector instead of a point light source. The image can be formed on a transparent glass window through the cooperation of a light source, digital micromirror device, and optical system to cure the entire pattern of 1 layer in a single exposure (Fig. 4A) [82].

Zhang et al. [83] developed a high-performance DLP-based tunable rapid printing technology that could print complex SMPs in the 30s while maintaining geometric accuracy (Fig. 4B). Invernizzi et al. [84] developed a thermally driven SMP with self-healing capability through DLP. Wu et al. [85], using polyethylene glycol (PEG) diacrylate as the main raw material, achieved different degrees of cross-linking of different parts of the component by controlling the light intensity distribution of the projector. The printed structure showed reversible shape changes and had great potential for application in vascular stents. Wang et al. [86] developed a novel DLP-based 4D printing technique to prepare near-infrared (NIR) photosensitive cardiac structures for myocardial regeneration. The shapes of the structures can be adjusted to match the aligned structure of the patient's myocardium. Tests showed that the 4D curved cardiac structures had uniform cell distribution and good myocardial maturation, demonstrating the great potential of DLP in tissue regeneration.

A higher-resolution printing method based on DLP, projection micro stereolithography (P*μ*SL), has been developed. Han et al. [87] achieved high-resolution printing of poly(N-isopropylacrylamide) using P*μ*SL. Danish et al. [88] investigated acrylic epoxidized soybean oil for 4D printing and explored the effect of 4D printing parameters (laser operating frequency and printing speed) on the different properties of printed materials for applications in various fields.

### Selective laser sintering

SLS is a 4D printing technology based on the powder bed fusion process. First proposed by C.R. Dechard in 1989, it is one of the most popular AM technologies. SLS uses 3D model data to selectively increase the temperature of a specific area in the powder bed using a laser. The solid powder in that area is sintered and bonded to the formed part as the temperature increases. When a layer of powder has been sintered, the powder bed is lowered by 1 thickness layer, and the powder deposition system adds a new layer of powder to the powder bed for the next sintering cycle. The steps are repeated to print the desired geometry (Fig. 4C) [89].

Wang et al. [90] investigated the powder-spreading process of SLS and analyzed the effect of powder spreading by spreaders with various geometries. Shi et al. [91] explored the effect of different powder material properties on the components prepared with SLS. Xin et al. [92] used experimental results to validate a multiphysics field model for the melting process of polymer powder beds, confirming the close relationship between the different physical phenomena in SLS and their contributions to the prediction of various parameters of the melting region. Wu et al. [93] prepared a magnetically driven fixture by SLS using neodymium–iron–boron (NdFeB) and thermoplastic PU. They tested its remote deformation under the action of an external magnetic field (Fig. 4D). Shuai et al. [94] prepared a porous bone stent by SLS, which exhibited excellent biocompatibility, promoted cell adhesion, and stimulated directional cell proliferation. Mei et al. [95] synthesized a polyamide-based thermoplastic polyamide elastomer using a “one-pot” melt polycondensation method and used freeze crushing to prepare the powders for SLS. Subsequently, they investigated the effects of various sintering parameters on the sintered parts and examined the shape memory properties of the thermoplastic polyamide elastomer sintered parts prepared by SLS.

### Multimaterial printing

As 4D printing technology has evolved, people have begun to combine multiple materials with increasingly sophisticated 4D printing technology to print multimaterial structures in a single pass. Multimaterial 4D printing provides a path to create digital materials with different *T*_*t*_ values, enabling the production of objects with more complex multishape and sequential transformations (Fig. 4E). The development of multimaterial 4D printing is the enabling technology for accelerating the growth of the smart material area. However, there are unresolved problems with multimaterial printing, such as limited material selection, low printing resolution, and low survival efficiency, and further research is needed to develop its application potential [96,97].

Lopes et al. [98] evaluated the effect of interfaces formed between the same and between different materials on the performance of multimaterial printed objects, demonstrating the importance of boundary design in multimaterial printing. Sossou et al. [99] proposed a voxel-based modeling framework for simulating the behavior of a wide range of materials. The work allows us to predict the process of shape change in smart materials, thus enabling the modeling and simulation of homogeneous and heterogeneous objects. Wu et al. [100] prepared a composite material by multimaterial printing using 3 materials with different *T*_*t*_. After the thermomechanical programming process, the composite could sequentially achieve different deformations as the temperature was increased. Ge et al. [47] introduced an automatic material conversion module in P*μ*SL to achieve the 4D printing of multimaterial SMPs able to sequentially deform (Fig. 4F). Mao et al. [101] proposed sequential self-folding structures prepared by multimaterial printing. The structures could exhibit a rapid response to temperature and be formed into a specified shape by controlled shape-changing sequences.

### Continuous fiber printing

In addition to the above 4D printing methods, continuous fiber 4D printing has recently started to gain attention. Most continuous fiber printing is currently based on FDM, and it has similar requirements to conventional processing, necessitating the careful consideration of the suitability and compatibility of fiber and matrix to reduce processing defects. Continuous fiber printing can generally be divided into 2 categories, one based on in situ or in-line impregnation methods using dry fiber bundles and the other based on the application of preimpregnated towpregs (Fig. 4G). The emergence of continuous fiber printing has enhanced the mechanical properties of 4D-printed devices to meet the need for large deformation and large load-bearing capacity while enabling controlled self-deformation of the device in response to external conditions. It has broad application prospects in aerospace, biology, and soft robotics [102].

Tian et al. [103] reported a fiber trajectory design method for 4D-printed composite structures. The shape and deformation processes of the composite structure were precisely controlled by controlling the fiber orientation. Dong et al. [104] proposed a novel strategy to fabricate electrically driven SMPCs and systematically investigated the mechanical and shape memory properties of the SMPCs. Zeng et al. [105] modified an FDM-based 3D printer with dual feed channels and fabricated a series of continuous carbon fiber-reinforced SMPCs. They explored the relationship between various printing parameters and the bending properties of 4D-printed SMPCs and investigated their electrothermal driving characteristics and shape memory behavior. Chen et al. [106] used a dual-nozzle 3D printer to print continuous carbon fiber-reinforced SMPCs with excellent mechanical and electrical properties. They investigated the effect of material parameters and applied voltage on the shape memory electrothermal behavior and demonstrated the potential of the SMPCs as a deployable gripper (Fig. 4H).

## 4D-Printed Structures

As the requirements for instruments in various fields become more demanding, people no longer focus only on improving the performance of the material themselves; they also investigate 4D-printed structures, which help their application in various situations. Below, the discussion will focus on some special structural designs.

### Biomimetic structures

**Fig. 5.**
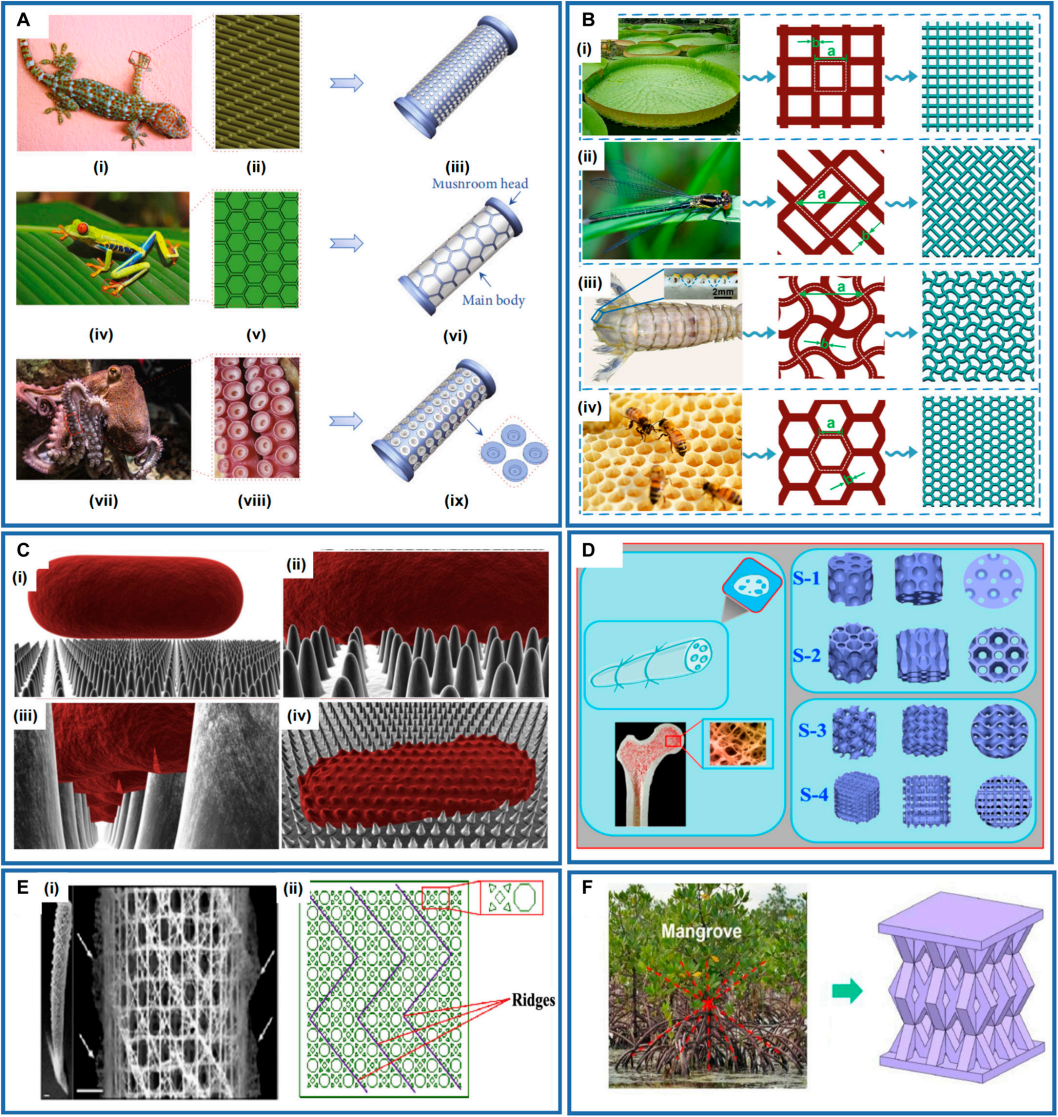
(A) Highly adhesive biomimetic stent: (i to iii) Gecko, (iv to vi) tree frog, and (vii to ix) octopus. Reproduced with permission from [107]. Copyright 2022 American Association for the Advancement of Science. (B) (i) Victoria Kreziana leaves, (ii) dragonfly wings, (iii) mantis shrimp telson, and (iv) honeycomb. Reproduced with permission from [114]. Copyright 2022 Elsevier. (C) The bactericidal mechanism of the superhydrophobic nanopillar structure on the surface of the cicada wing: (i) cells in contact with the nanopillar structure, (ii) cells adsorbed on the nanopillar, (iii) the outer layer of the cell begins to break down, and (iv) cells collapsed on the surface. Reproduced with permission from [115]. Copyright 2013 Elsevier. (D) The porous stent structure inspired by lotus rhizome structure and bone trabecular structure. Reproduced with permission from [117]. Copyright 2021 Elsevier. (E) (i) Photograph of a glass sponge and the fragment of the cage structure and (ii) plane graph of the deployable structure and cell element of bioinspired tracheal stent. Reproduced with permission from [118]. Copyright 2019 Elsevier. (F) 4D printing cartilage stent inspired by mangrove structure. Reproduced with permission from [119]. Copyright 2023 American Chemical Society.

Biomimetic structures are constructed by studying the structure of biological bodies and mimicking those to achieve similar functions and are mainly used in the biomedical field. Biomimetic structures are often complex and difficult to prepare through traditional manufacturing methods. 4D printing technology allows biomimetic manufacturing to develop rapidly in the biomedical field [107]. Studying highly adhesive biological structures in nature has given researchers many useful insights. For example, the gecko’s foot features numerous bristles that prove a high adhesion capacity through van der Waals’ forces between the bristles and the surface of the object [108]. When the van der Waals’ forces introduced by the structure are insufficient to support the object's weight, the bristle structure of the gecko foot will increase its adhesion capacity as sliding proceeds until a certain upper limit is reached [109]. Like gecko feet, tree frogs also have unique microstructures on their toe pads. Concave-tip nanopillars have been identified on the polygonal epithelium cells of the tree frog toe pad, which provide adhesion mainly through capillary action. The nanopillar structure of tree frog toe pads maintains a high adhesion ability on damp surfaces, unlike the bristle structure of gecko feet, whose adhesion ability significantly decreases when wet [110]. Studies of the structure of octopus suction cups showed that octopus suction cups comprise a funnel above and an acetabulum below held on the surface by the gaps created by the collapse of the structure, allowing them to adhere well under dry and wet conditions [111]. In addition, mussels, mother-of-pearl, spiders, beetles, and amphibians have highly adhesive biological structures. Highly adhesive biomimetic structures can be applied to biological stents (Fig. 5A) to effectively improve the migration resistance of stents by up to 470% compared to stents without surface microstructures [107,112,113].

Beyond the use of biomimetic structures to enhance the migration resistance of stents, many biological structures have been investigated to improve the properties of medical instruments. For example, Deng et al. [114] introduced biomimetic structures inspired by the *Victoria cruziana* leaf, dragonfly wing, mantis shrimp telson, and honeycomb into 4D-printed orbital stents to optimize their mechanical properties (Fig. 5B). Pogodin et al. [115] developed a superhydrophobic nanopillar structure that mimicked the surface of cicada wings and investigated the bactericidal mechanism of the biomimetic structure, which can effectively reduce bacterial contamination of medical instruments (Fig. 5C). Jiang et al. [116], inspired by the layered structure of the lotus leaf surface, prepared biomimetic structures with advanced synergistic antibacterial properties and long-term mechanical bactericidal activity. Zhao et al. [117] investigated the structure of lotus rootstock. They used it to prepare porous bone tissue stents with low density, high porosity, and low flow resistance, facilitating nutrient exchange (Fig. 5D). Zhao et al. [118] introduced a glass sponge structure to the tracheal stent to enhance the strength, stability, and adaptability of the stent (Fig. 5E). Deng et al. [119] found that the unique structure of mangroves enabled them to grip the ground stably and reduce the formation of wind and waves effectively, exhibiting excellent mechanical properties. Therefore, they designed the structural unit of a cartilage stent based on it (Fig. 5F). Inspired by collagen fibers, Lin et al. [120] introduced a wave-like microstructure into a left atrial appendage occluder (LAAO). Introducing this biomimetic structure enabled the left atrial appendage occluder to have a “J”-shaped stress–strain curve resembling that of biological tissues, which exhibits synergistic deformation with the tissues and can protect the tissue and effectively reduce the risk of failure of medical instruments. In addition, microstructures inspired by various biological structures have been developed to facilitate cell growth. They have been applied to many medical instruments, such as bone tissue and vascular stents, contributing to their improved performance in the biomedical field [121–123].

### Negative Poisson's ratio structures

**Fig. 6.**
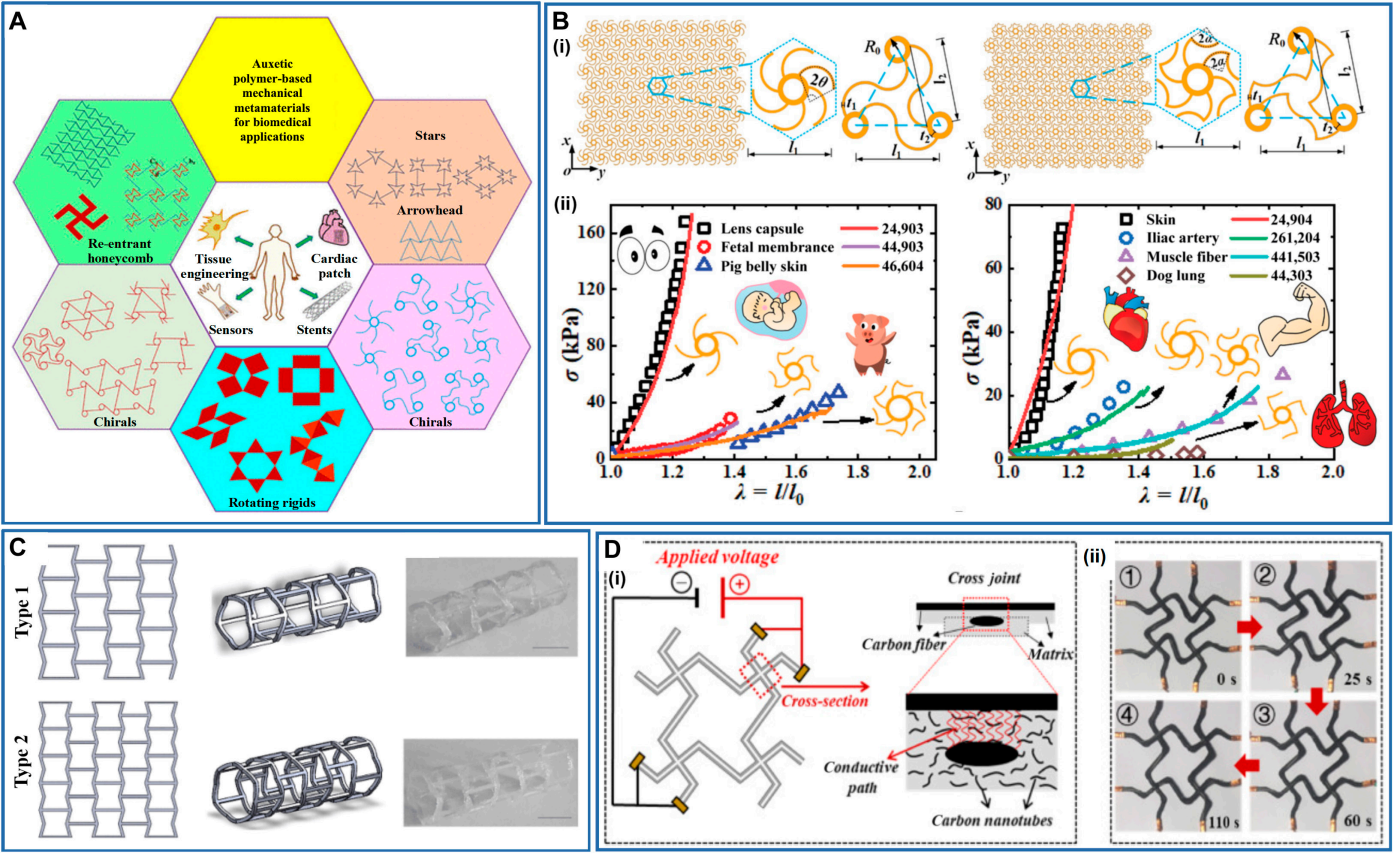
(A) Negative Poisson's ratio structures for biomedical applications. Reproduced with permission from [126]. Copyright 2022 American Chemical Society. (B) (i) Schematic of novel chiral metamaterials and (ii) comparison of stress–strain curves between metamaterials and tissues/organ. Reproduced with permission from [128]. Copyright 2020 Wiley-VCH. (C) Vascular stents with negative Poisson's ratio effect. Reproduced with permission from [129]. Copyright 2020 Springer Nature. (D) (i) The cross-sectional microstructure of electrically driven continuous fiber reinforced SMPC with negative Poisson's ratio effect and (ii) electro-induced behavior of the negative Poisson's ratio structure. Reproduced with permission from [104]. Copyright 2021 Elsevier.

Metamaterials are materials that exhibit extraordinary physical properties that natural materials do not possess, and these properties are mainly derived from special artificial structures [124,125]. Among the many special structures of metamaterials, those with a negative Poisson’s ratio effect are often used in the 4D printing of biomedical instruments (Fig. 6A) [126]. These structures experience lateral expansion (contraction) when subjected to external longitudinal tension (compression) and have excellent mechanical properties. Based on the deformation mechanism of negative Poisson’s ratio structures, they can exhibit several structures, including inner concave polygon structures, rotating rigid body structures, chiral structures, perforated plate structures, and node–fiber structures.

Negative Poisson’s ratio structures have many applications in the biomedical field. For example, Ahn et al. [127] introduced negative Poisson’s ratio structures in multilayer tubular vascular stents to improve compliance. Xin et al. [128] developed a novel editable and tunable chiral structure stent prepared by 4D printing. They found that the introduction of the chiral structure allowed the stent to mimic the J-shaped stress–strain curve of a specific tissue/organ and could be programmed to convert to another biomaterial, demonstrating the great potential of negative Poisson’s ratio structure for biomedical applications (Fig. 6B). Lin et al. [129] developed personalized vascular stents with negative Poisson’s ratio structures by 4D printing and optimized their structures using a genetic algorithm. The stents exhibited excellent shape memory properties, and they could dilate narrow vessels within 5 s (Fig. 6C). Lei et al. [130] designed a new type of negative Poisson’s ratio structure, and the mechanical properties of the structure could be programmed, erased, and reprogrammed. They anticipated it being used to simplify the manufacture of cardiovascular stents and similar deployable structures. In addition to the biomedical field, negative Poisson's ratio structures have also been studied in other fields. Dong et al. [104] developed an electrically driven negative Poisson's ratio structure through continuous fiber 4D printing technology. The SMPC can undergo self-expanding shape changes upon electrical stimulation and has the potential to serve as a key component of deployable trusses, adaptive energy absorption devices, or other lightweight smart systems (Fig. 6D).

### Origami structures

Origami structures have also received significant attention in 4D printing due to their novel design methods and unique deformation modes along the crease lines. Origami structures show excellent flexibility due to their unique structural properties. Origami structures can be used to fold 2D structures into 3D structures, which have the advantages of lightness, rapid manufacturing, large deformation, and customizability.

**Fig. 7.**
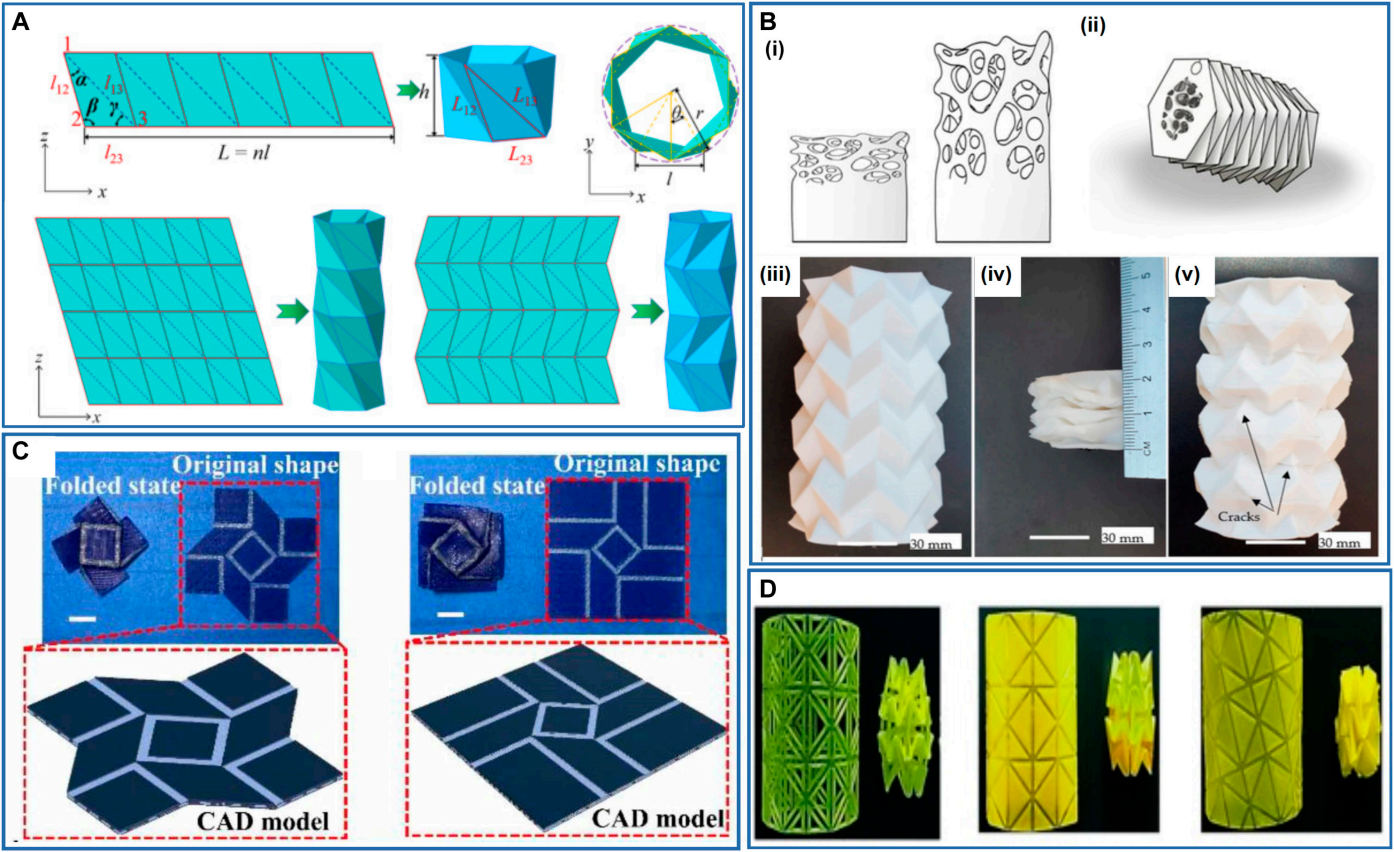
(A) The triangular cylindrical origami structure and the panel with crease patterns consisting of mountain crease lines (solid lines) and valley crease lines (dotted line). Reproduced with permission from [131]. Copyright 2020 Elsevier. (B) (i) Natural cancellous bone, (ii) combined tessellation origami and natural cancellous bone design, (iii) 4D-printed origami structure, (iv) the origami structure after compression, and (v) the origami structure after recovery. Reproduced with permission from [131]. Copyright 2021 Multidisciplinary Digital Publishing Institute. (C) Folded states, original states, and computer aided design (CAD) models of the origami-inspired self-deployment sandwich structures. Reproduced with permission from [134]. Copyright 2020 IOP Publishing. (D) Stents with origami structure prepared via 4D printing before and after folding. Reproduced with permission from [135]. Copyright 2022 Elsevier.

These properties of origami structures have led several researchers to investigate their potential uses. Tao et al. [131] investigated 4D-printed origami structures with adjustable stress–strain curves and compression twist deformations (Fig. 7A). The folding deformation of the structures provides them with good impact resistance and energy absorption, and the mechanical properties of the structure can be further adjusted by controlling the temperature. Wan et al. [132] developed Tachi–Miura polyhedron origami structures with 4D printing technology. The test results indicate that the structure can exhibit different mechanical properties and deformation modes by adjusting the structural parameters. Langford et al. [133] used PLA for the 4D printing of functional stents with origami structures that can be used in minimally invasive orthopedic surgery. When the stent reaches its target location, it undergoes shape recovery, allowing it to increase in size and fill the cavity, replacing the missing extracellular matrix in the tissue to temporarily support structural integrity and coordinate cellular activity (Fig. 7B). Xin et al. [134] developed thermally driven origami-inspired self-deployment sandwich structures with large area change ratios and demonstrated their excellent mechanical properties and area change ratios (Fig. 7C). Zhao et al. [135] proposed and demonstrated origami-derived self-assembly stents that can be folded into small shapes along predesigned creases to complete the self-assembly process when heated (Fig. 7D).

### Other structures

In addition to the above structures, some other special structures are also used in 4D printing. These special structures enable 4D-printed objects to obtain some extraordinary properties that allow them to be used in special environments, advancing the development of 4D printing technology.

**Fig. 8.**
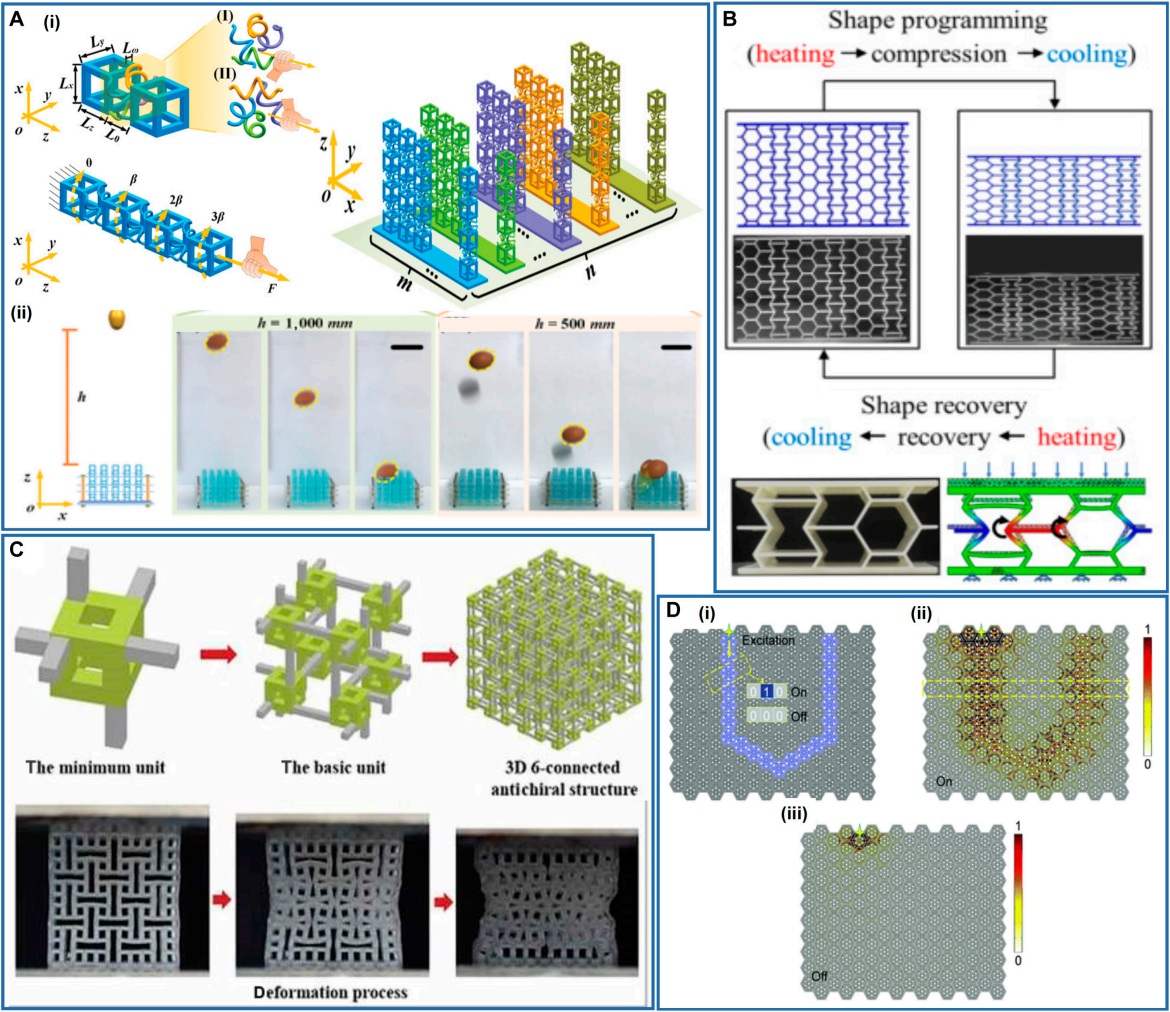
(A) (i) The CAD models of the mechanical pixel and the pixel mechanical metamaterial. (ii) The dynamic impact experiment of free-falling eggs on the pixel mechanical metamaterial. Reproduced with permission from [137]. Copyright 2022 Wiley-VCH. (B) Shape memory process of zero-Poisson's ratio structures. Reproduced with permission from [138]. Copyright 2020 Elsevier. (C) The evolution illustration of the 3D lightweight meta-architecture and its deformation process. Reproduced with permission from [125]. Copyright 2022 Taylor & Francis Group. (D) (i) Programmable “U” shape propagation path of the 4Dprinted structure, (ii) the structure at the “On” state, and (iii) the structure at the “Off” state. Reproduced with permission from [141]. Copyright 2021 Royal Society of Chemistry.

Tao et al. [136] developed a multistable 4D-printed metamaterial structure with controllable deformation and recovery, deployable function, adjustable mechanics, and reusability, enabling controllable changes in the shape, rigidity, and function of 4D-printed objects in time and space dimensions to meet the requirements of applications in different settings. Xin et al. [137] proposed a 4D pixel mechanical metamaterial structure, which exhibited the tunability, programmability, and reconfigurability of its mechanical behaviors (Fig. 8A). The structure eliminates the internal coupling constraints and demonstrates great deformation potential, showing great potential for information encryption, kinematics controllers, and buffer devices. Liu et al. [138] created a 4D-printed structure with a zero Poisson's ratio that could automatically adjust stiffness, energy absorption, and vibration isolation capability with temperature changes and exhibited excellent low-frequency vibration isolation without causing stiffness or strength degradation (Fig. 8B). Zhao et al. [139] designed a composite structure with a 2-way SME. The Poisson's ratio of the structure could change with the structural parameters and could even be converted between positive and negative. Xue et al. [140] designed a surface relief structure and used it for temperature-controlled transfer printing. Their research results contribute to the application of SMP in electronics and reversible adhesion. Zhang et al. [125] designed a 3D lightweight meta-architecture consisting of 6 connected antichiral honeycombs (Fig. 8C). They achieved the programmability of the mechanical properties of the structure by adjusting their structural parameters, so the structure can achieve greater ductility under compression while retaining relatively high strength and low density. Li et al. [141] studied a 4D-printed structure for self-adaptive bandgap switching and programmable elastic-wave propagation paths. By controlling the shape change of the 4D-printed structure, active elastic-wave guiding and programming of propagation paths could be realized (Fig. 8D).

## Actuation Methods of 4D-Printed Structures

**Fig. 9.**
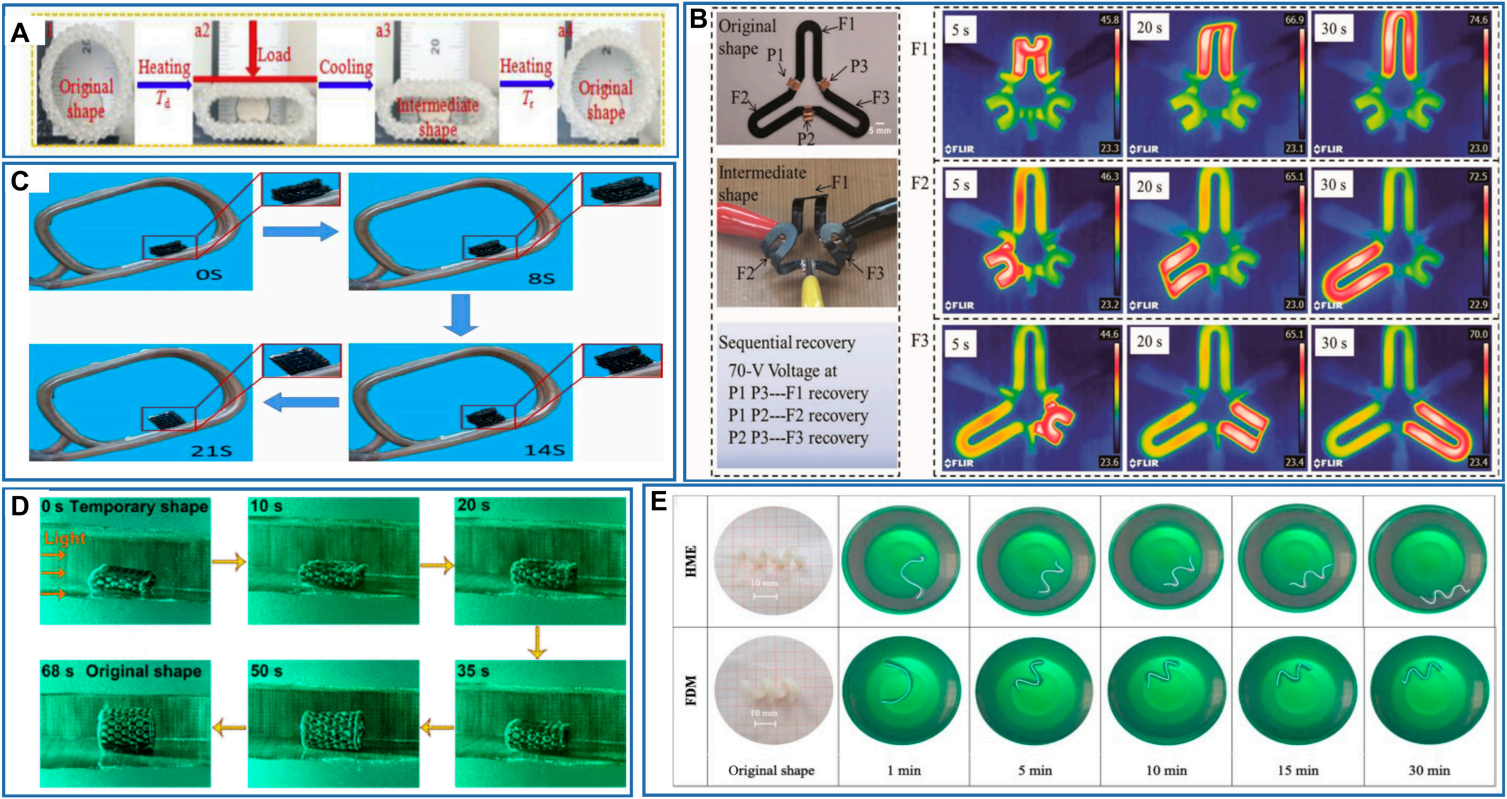
(A) The shape recovery process of 4D-printed structure under the action of temperature. Reproduced with permission from [144]. Copyright 2018 Elsevier. (B) The shape recovery process of 4D-printed structure under the action of current. Reproduced with permission from [146]. Copyright 2019 Wiley-VCH. (C) The shape recovery process of 4D-printed structure under the action of a magnetic field. Reproduced with permission from [150]. Copyright 2022 IOP Publishing. (D) The shape recovery process of 4D-printed structure under the action of light. Reproduced with permission from [153]. Copyright 2022 American Chemical Society. (E) The shape recovery process of 4D-printed structure under the action of water. Reproduced with permission from [158]. Copyright 2019 Elsevier.

The thermal drive is the classic and best-described actuation method for 4D-printed structures. However, as the applications and performance requirements for 4D-printed structures in various fields expand, thermally driven 4D-printed structures are unemployable in certain scenarios, as shown in Table. Consequently, to meet the requirements for precise control of 4D-printed structures in various situations and facilitate better applicability in various fields, other actuation methods have been developed, such as electric, magnetic, optical, water, or solutions (Fig. 9).

**Table.** Research progress of 4D-printed SMPs in various fields.

Application	Materials	Actuation methods	Printing method	Refs.
Vascular stent	PLA/Fe_3_O_4_	Magnetic drive	DIW	[72]
Bone tissue engineering	PLA/Fe_3_O_4_	Magnetic drive	FDM	[162]
	PU/nHA	Thermal drive	DIW	[119]
Occluder	PEG/PLA/BaSO_4_	Thermal drive	FDM	[163]
Tracheal stent	PCL	Thermal drive	SLA	[164]
	PLA/ Fe_3_O_4_	Magnetic drive	FDM	[165]
Orbital stent	AuNPs/nHA/PU	Thermal drive	DIW	[114]
Intestinal stent	PEG/PLA	Thermal drive	FDM	[166]
Solar-panel array	FLX9895,RGD835	Thermal drive	PolyJet	[167]
Antenna	PLA	Thermal drive	FDM	[134]
Deployable boom	PLA/MWCNTs	Thermal drive	FDM	[168]
Morphing wing flap	VeroClear	Electrical drive	Inkjet printing	[31]
Interactive Christmas tree and self-folding robot	PLA	Thermal drive	FDM	[169]
Conductive element	ESBO/BFDGE/CNF	Thermal drive	DIW	[170]
Temperature sensor	PCL	Thermal drive	SLA	[80]
Multitemperature sensing electrical safety device	Tert-butyl acrylate/PEGDA(or PEGDMA)/Cu	Thermal drive	DLP	[171]
Soft robot	LCE	Electrical drive	DIW	[172]
	PLA/CB	Thermal drive	FDM	[173]
	Methacrylate	Thermal drive	P*μ*SL	[47]
	PEU	Thermal drive	FDM	[174]
Greenhouse covering device	VeroClear	Thermal drive	TPL	[48]
Smart textile	PLA	Thermal drive	FDM	[175]
Food	Purple sweet potato purees	Microwave dehydration	DIW	[176]
Smart wind turbine blade	PLA	Thermal drive	FDM	[177]

### Thermal drive

The thermal drive controls the glass transition through heat transfer from the thermal medium to achieve the SME. The thermal drive of 4D-printed structures has been studied in detail. Liu et al. [142] printed a 4D-printed laminated strip by FDM and achieved its sequential shape-morphing behavior under heating conditions at 90 °C. Wang et al. [143] introduced a novel strategy including 4D printing, UV postcuring, and thermal curing and used it to form triple SMPs with high strength and fracture toughness. They have used this strategy to print a series of 4D-printed structures to demonstrate their good processing performance. Zhang et al. [144] prepared circular braided tube preforms and their silicone elastomer matrix composites by 4D printing and demonstrated their shape recovery behavior under heating conditions. Zhang et al. [145] used a novel SMP with excellent shape memory properties to print a vascular stent. The stent can undergo shape recovery in the near-body temperature range, making it suitable for intracorporeal implantation.

### Electrical drive

The electrical drive is an actuation method developed based on thermally driven SMPs. It is achieved by adding substances with conductive properties, such as carbon nanotubes (CNTs), conductive carbon black (CB), graphene, and metal nanoparticles, to thermally driven SMPs. When an electric current passes through this SMPC, the Joule heating generated by the electric energy causes the temperature of the material system to increase, resulting in an SME of the 4D-printed structures.

There has been some exploration of electrical drives for 4D-printed structures. Liu et al. [146] prepared an electroactivated 3-pronged device using CNT-reinforced PLA by a 4D printing technique, and the 4D-printed SMPC exhibited sequentially controlled shape memory behavior under an applied dc voltage. Dong et al. [147] fabricated various 4D-printed structures based on PLA/CNT and investigated their electrical, thermal, and shape memory properties. The shape recovery ratio of these 4D-printed SMPCs reached more than 90% under a certain voltage, and they are expected to be used to prepare electroactive deformable devices. Wei et al. [148] designed a nanocomposite using silver-coated carbon nanofibers (Ag@CNFs) as conductive fillers to prepare an electrically driven smart gripper. Zhang et al. [149] developed an innovative 4D printing strategy to print electrically driven composites with sandwich structures. They printed a deployable and expandable structure based on this strategy that mimicked a bat wing and exhibited shape recovery behavior under current stimulation.

### Magnetic drive

Similar to electrical drive, magnetic drive is also an actuation method developed based on thermally driven SMPs. Magnetic particles (such as *γ*-Fe_2_O_3_, Fe_3_O_4_, NdFeB, carbonyl Fe, and Ni-Zn ferrite) are incorporated in a thermally driven SMP, thus giving it the ability to achieve shape memory through magnetic fields. When an alternating magnetic field is applied to SMPCs containing magnetic particles, these particles will convert electromagnetic energy into thermal energy through a loss mechanism and conduct thermal energy to the surrounding thermally driven SMP matrix, thus activating the SME of the 4D-printed structures.

Several studies have been conducted on magnetically driven SMPCs. Zhou et al. [150] prepared a 4D-printed PLA/Fe_3_O_4_ stent that can be recovered to working size and shape with an alternating magnetic field to fill a bone defect site. Zhu et al. [151] developed a poly(dimethylsiloxane)/Fe (PDMS/Fe) composite ink to print magnetically driven 4D structures. They demonstrated the rapid response of the 4D-printed structures under an external magnetic field. Huang et al. [152] printed a magnetically driven soybean oil-based vascular stent with an inner concave hexagonal negative Poisson’s ratio structure by DLP, and the stent exhibited more than 90% shape recovery and shape fixation ratio under an external magnetic field.

### Light drive

Light-driven SMPCs can be achieved by filling the thermally driven SMP with photothermal particles (e.g., nanogold [AuNPs], CB, graphene oxide, or black phosphorus particles). Under the action of NIR irradiation, the internal particles in SMPCs generate heat through a photothermal conversion effect, thereby producing SME in the 4D-printed structures.

Deng et al. [153] prepared a series of 4D-printed structures with light-driven SME by adding AuNPs to a shape memory PU (SMPU) matrix through secondary dissolution. These 4D-printed structures exhibited shape recovery under the action of light. Cui et al. [154] developed a novel NIR-responsive nanocomposite and used it to prepare 4D-printed structures. The NIR-sensitive 4D transformation behavior of the 4D-printed structures resulted in remote, precise, and dynamic control in both time and position. Wang et al. [155] prepared a photothermally responsive bone tissue engineering stent by combining black phosphorus nanosheets and osteogenic peptides into *β*-tricalcium phosphate/poly(lactic acid-co-trimethylene carbonate) via 4D printing. The SMPC exhibited shape recovery behavior in the presence of NIR light with a recovery ratio of 90%.

### Water drive or solution drive

The concept of using water to drive SMP was first proposed by Huang et al. in 2005 [156,157]. They found that the shape recovery of deformed PU wires would occur after immersing them in water for 30 min at room temperature. This actuation method was shown to differ from the conventional actuation method in that the transition temperature of the SMP is modified to produce SME instead of overall temperature regulation. When water molecules enter the molecular chains of SMPs, the intermolecular interactions are weakened by reducing the hydrogen bonds between the molecular chains. This results in a decrease in the *T*_*t*_ of the SMP, which allows the SMP to undergo shape recovery even at a constant temperature. Further research showed that SME can also be achieved with certain organic solvents.

The water-driven or solution-driven method, which can produce SMEs without a heating process or chemical reactions, has attracted the attention of many researchers as a new direction worthy of further exploration. Melocchi et al. [158] reported the fabrication of a 4D-printed water-driven PVA-based intravesical drug delivery system using FDM and hot melt extrusion. The spiral-shaped device can undergo shape recovery at 37 °C under the driving effect of water, and the recovery process is relatively fast. Later, their team [159] developed a novel 4D-printed structure that could be driven in HCl, using it as a scalable gastric retention drug delivery system. Han et al. [160] prepared a novel microneedle (MN) with a bioinspired reverse curved barb structure by P*μ*SL. When the MN is immersed in ethanol, the horizontally printed barbs on MN deform to a backward-facing shape, enhancing its tissue adhesion ability.

## Applications

### Biomedical

**Fig. 10.**
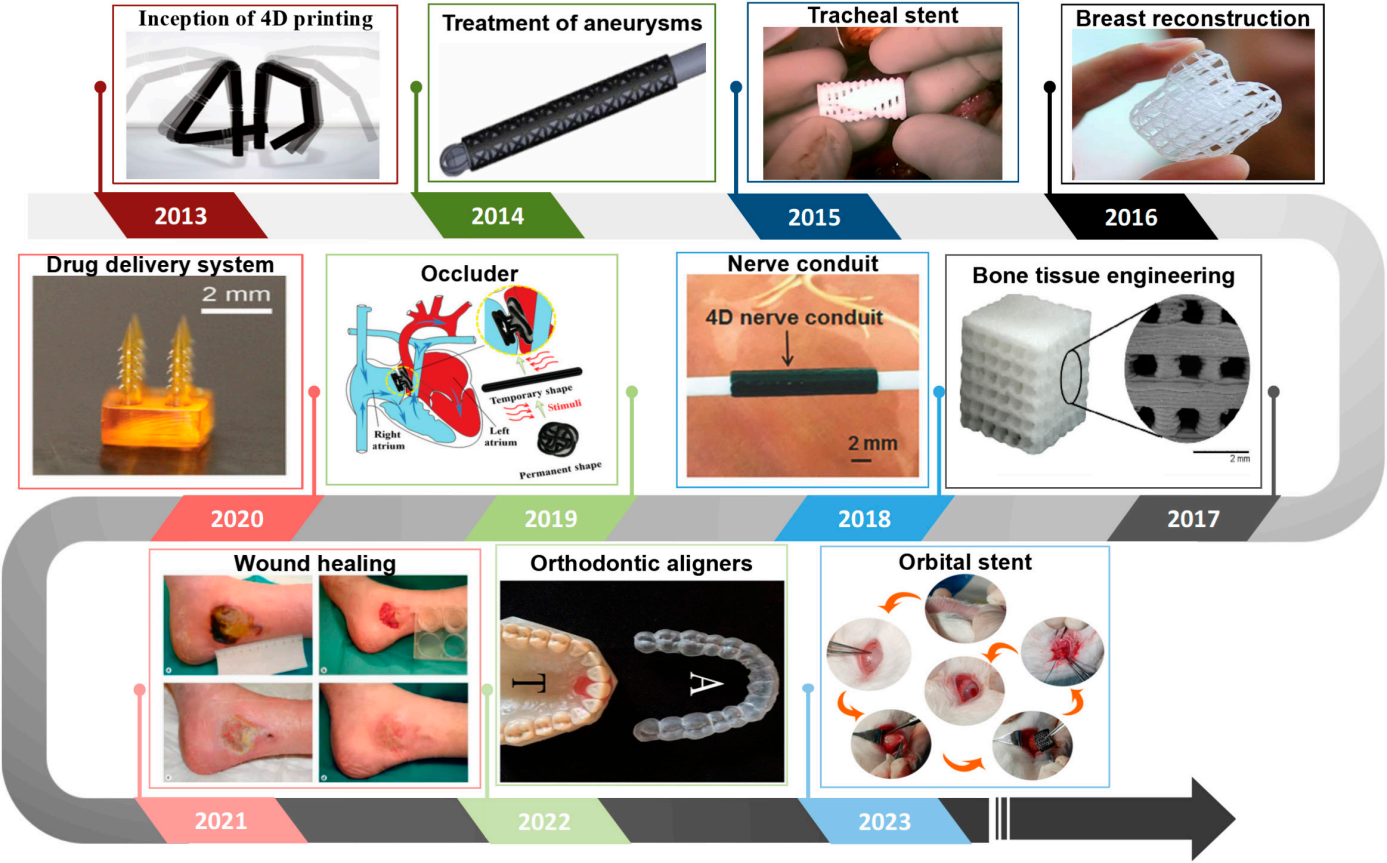
Year-by-year development of 4D-printed SMPs in biomedical applications.

In recent years, with the increasing demand for minimally invasive technologies in clinical care, 4D printing technologies that can shape SMPs and SMPCs into complex structures have received much attention in biomedical applications (Fig. 10) [161]. In the following sections, I will introduce several major recent research advances in 4D-printed SMPs and SMPCs for biomedical applications.

**Fig. 11.**
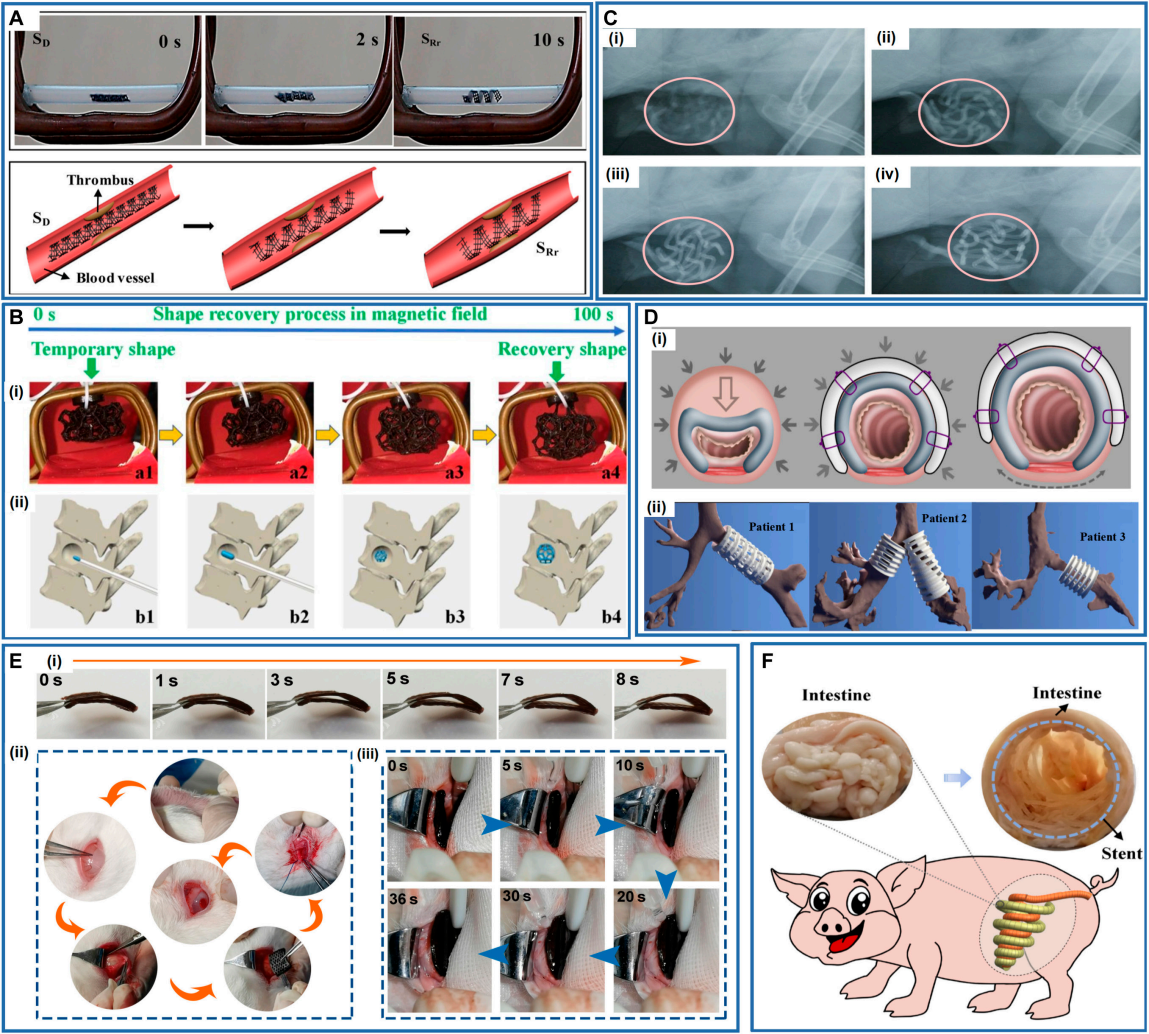
(A) Shape recovery process of 4D-printed stent under alternating magnetic field. Reproduced with permission from [72]. Copyright 2017 American Chemical Society. (B) (i) Shape recovery behavior of 4D-printed stent in magnetic field and (ii) simulation of the mechanism of 4D-printed stent. Reproduced with permission from [162]. Copyright 2019 Elsevier. (C) In vivo overall radiopacity examination by implanting VSD occluders. Reproduced with permission from [163]. Copyright 2023 Wiley-VCH. (D) (i) Mechanism of 4D-printed tracheal stents in biomedicine and (ii) 4D-printed tracheal stents on a virtual assessment of applicability. Reproduced with permission from [164]. Copyright 2015 American Association for the Advancement of Science. (E) (i) Shape recovery process of the stent, (ii) the implantation procedure of the orbital stent, and (iii) shape recovery process of the stent in the orbit of the rabbit. Reproduced with permission from [114]. Copyright 2022 Elsevier. (F) Feasibility of the 4D-printed shape memory intestinal stent opening simulated obstructed swine intestine. Reproduced with permission from [166]. Copyright 2023 Elsevier.

Wei et al. [72] designed a vascular stent that could be remotely driven by a controlled magnetic field prepared by DIW printing with UV cross-linked PLA/Fe_3_O_4_ composite ink. To examine the shape memory performance of this vascular stent, they placed a vascular stent with a temporary shape into a plastic tube holder, and the inner diameter of the vascular stent changed from 1 to 2.7 mm in 10 s under the effect of an alternating magnetic field (Fig. 11A).

Zhang et al. [162] added PLA/Fe_3_O_4_ composite filaments to an FDM printer to 4D print a porous stent for personalized bone repair that could recover its initial shape within 100 s under the effect of a magnetic field (Fig. 11B) and can be custom printed for different defect sizes.

Deng et al. [119] prepared an SMPC by adding nanohydroxyapatite (nHA) to a SMPU matrix, and a cartilage stent with a mangrove-inspired structure was prepared for cartilage defects by 4D printing. This stent showed good cytocompatibility, histocompatibility, and mechanical and shape memory properties. It could completely recover its shape within 60 s at human body temperature, providing a novel approach for personalized and minimally invasive treatment of cartilage defects.

Lin et al. [163] developed a radiopaque, biodegradable, and dynamically reconfigurable 4D-printed ventricular septal defect (VSD) occluder. They introduced barium sulfate as a radiopaque filler into PEG and PLA to prepare an SMPC that can undergo shape deformation at near-body temperature and printed a VSD occluder with a wavy biomimetic structure by FDM. The overall radiopacity of the occluder prevents the inaccurate localization that may occur when only a small number of radiopaque markers are incorporated in the alloy occluder and facilitates accurate intraoperative localization and postoperative follow-up monitoring (Fig. 11C). The application of this manufacturing process can be extended to prepare other smart medical devices, including various tissue engineering stents and drug delivery devices, opening the way for the rapid manufacturing of custom smart medical devices.

Morrison et al. [164] prepared a PCL-based tracheal stent using SLA and used it to treat tracheobronchomalacia. The 3D-printed tracheal stents were implanted in 3 patients with severe tracheobronchomalacia without adverse effects or complications, successfully treating the tracheal disease in these patients. In vivo, trials showed that the tracheal stent expanded as the airway grew, and it biodegraded over time in the body. The stents can, therefore, not only meet the personalized requirements of patients, but they are also biodegradable when the patient’s trachea grows healthy 3 years later, avoiding multiple surgeries that cause patients pain (Fig. 11D).

Zhang et al. [165] used FDM to prepare a shape memory PLA/Fe_3_O_4_ composite tracheal stent that could perform remotely controlled shape recovery by a magnetic field within 40 s with a shape recovery ratio of more than 99%. In addition, they used a curved rectangle as the basic structural unit of the stent, which allowed the stent to minimize stress concentration and maintain structural stability. By changing the curvature of the curved rectangle, Poisson’s ratio of the structural units could be adjusted. The stent could thereby be adjusted according to the condition of the patient, making it more targeted.

Deng et al. [114] doped AuNPs and nHA into SMPU to prepare 4D-printed orbital stents with excellent computed tomography development properties and shape memory properties, which were fabricated by 3D-Bioplotter to treat enophthalmic invagination (Fig. 11E). The 4D-printed stents incorporated a biomimetic honeycomb structure, which conferred good mechanical properties to support orbital tissues. In addition, the stent had good biocompatibility and shape memory and was able to recover its shape in water at 44 °C within 8 s. After implantation of the 4D-printed stent into the orbit of rabbits via minimally invasive surgery, the stent successfully recovered from its compressed, temporary shape to its initial shape under thermal stimulation, indicating its potential as an advanced medical device for personalized treatment in ophthalmology.

Lin et al. [166] developed a PEG/PLA-based 4D-printed shape memory biocomposite by melt extrusion and printed it by FDM to produce an intestinal stent with a wave-like network. The biocomposite exhibited stress-strain behavior similar to that of biological tissues, thus effectively reducing the risk of tissue damage. The stent had the advantages of high flexibility, biodegradability, biocompatibility, customized configuration, and the ability to complete the shape memory process at near-body temperature (42 to 43 °C). Furthermore, they successfully used the 4D-printed intestinal stent to open a simulated obstructed porcine intestine and recover the size of the intestinal lumen (Fig. 11F).

### Aerospace

In the aerospace field, traditional structures have inherent drawbacks such as high weight, high cost, and large deployable impact effects, which take up much space and thus reduce the effectiveness of aerospace missions. Devices made of shape memory materials can be compressed and packaged to reduce the amount of space they take up, then deployed after being transported to a designated location, thus solving the shortcomings of traditional structures; consequently, they have received considerable attention. Compared with other shape memory materials, SMPs, and their composites are lower density and have higher deformability, further expanding their advantages in the aerospace field. They are currently used in solar panel arrays, booms, hinges, antennas, and many other applications.

**Fig. 12.**
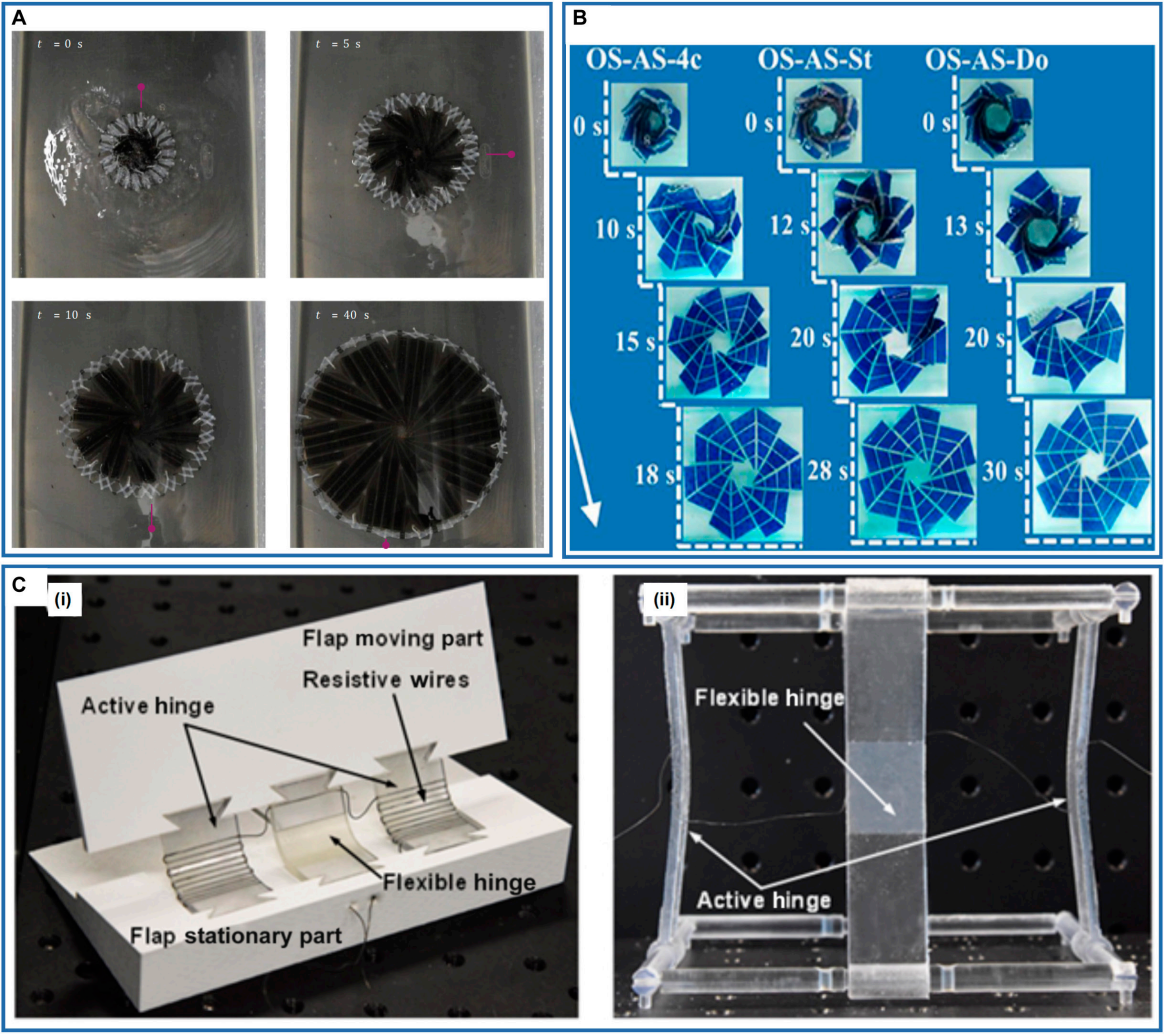
(A) The autonomous deployment of the solar-panel array. Reproduced with permission from [167]. Copyright 2019 American Physical Society. (B) The shape memory recovery process of origami-inspired structures. Reproduced with permission from [134]. Copyright 2020 IOP Publishing. (C) As-fabricated configuration of the 4D-printed structures: (i) morphing wing flap and (ii) deployable structure. Reproduced with permission from [31]. Copyright 2018 IOP Publishing.

Chen et al. [167] proposed a self-deployable system with a rotational periodicity inspired by the opening and closing mechanism of scissors. They prepared an autonomous solar panel array based on the system. They used SMP to fabricate both the origami substrate and the hubs in the scissor mechanisms by 3D printing, thus enabling the self-deployment of solar panel arrays in response to changes in ambient temperature. Tests showed that the system could achieve an expansion ratio of 1,000% in under 40 s (Fig. 12A), and the excellent expansion performance allows a spacecraft to accommodate a larger number of solar panels.

Xin et al. [134] proposed a 4D-printed origami structure with foldability and excellent mechanical properties, using various negative Poisson's ratio metamaterials as its interlayers to further improve the mechanical and shape memory properties of the origami. They printed these sandwich structures by FDM and tested their tensile properties, 3-point bending properties, and shape recovery properties, showing the fast response, large deformation, and excellent self-deployment capabilities of the origami structure (Fig. 12B), which has promising applications in space-deployable structures.

Herath et al. [168] prepared a 4D-printed deployable boom for small spacecraft by FDM using SMP and multiwalled CNTs (MWCNTs). The introduction of MWCNTs enables the light activation of the deployable boom, making it more suitable for aerospace. The deployable boom exhibits good compression strength and impact energy absorption with ~86 % shape recovery, but its dimensional stability and structural properties need further improvement.

Akbari et al. [31] prepared shape memory structures by a multimaterial inkjet 3D printing technique consisting of active and flexible hinges. The active hinges printed from SMP can fix the structure into a temporary shape after thermomechanical programming. The flexible hinges printed from elastomer can further increase the shape recovery rate and load-carrying capacity of the printed structure. They also embedded resistive wires in the structure to achieve localized Joule heating. They used this as the basis for designing electrically driven morphing wing flaps and deployable structures (Fig. 12C). The SMPCs exhibit excellent shape memory performance under electric drive and are expected to be used in aerospace applications.

### Electronics

Due to the limitations of traditional processing methods, electronic components can usually only be printed on flat substrates or processed into static 2-dimensional structures, which limits their development and application. The emergence of 4D printing technology has broken the limitations of traditional electronic components and promoted the development of smart deformable electronic components. 4D printing technology makes electronic components structurally designable and deformable, allowing them to be used in more advanced high-tech industries.

**Fig. 13.**
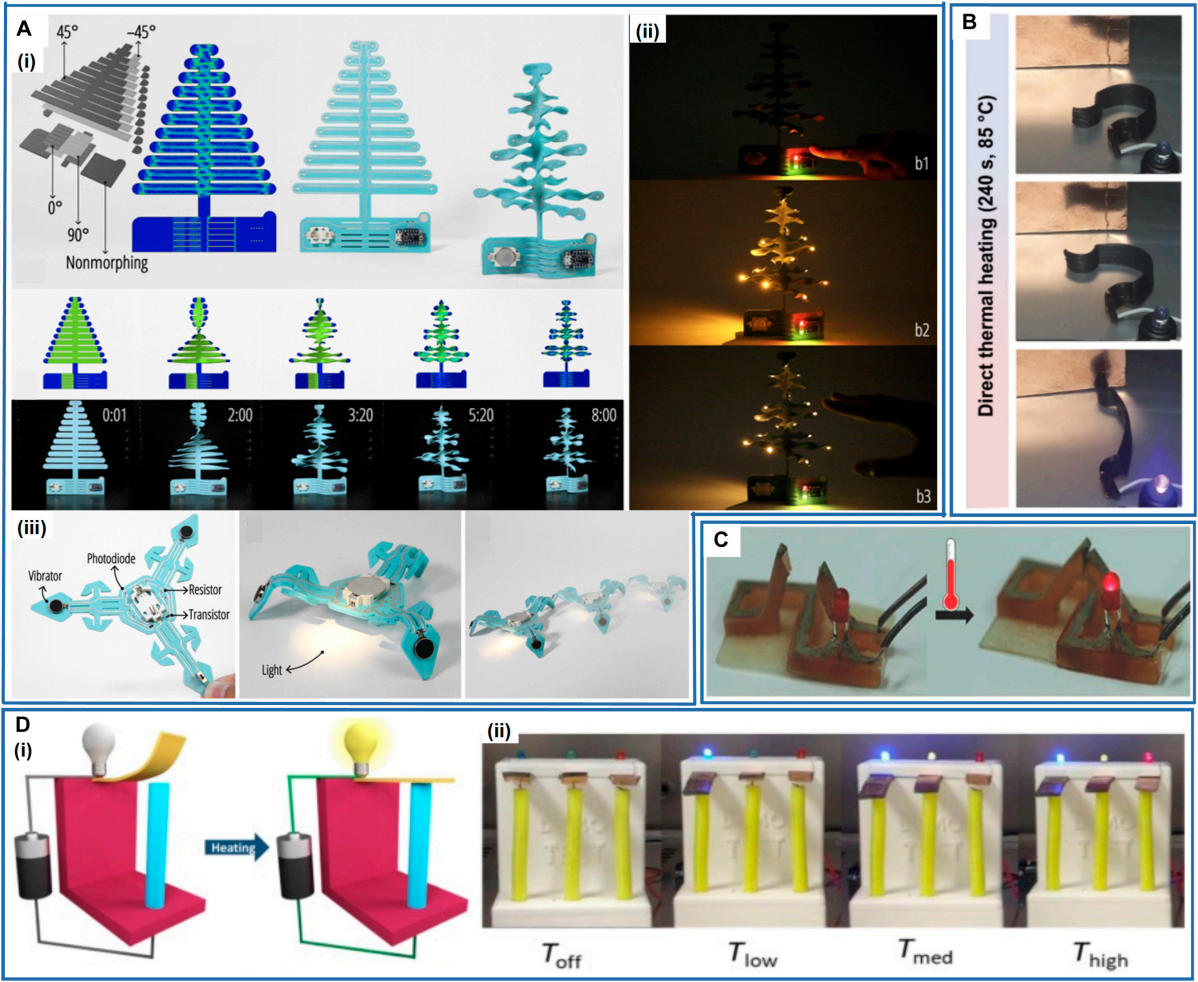
(A) (i) Self-morphing Christmas tree. (ii) The interaction scenario of the Christmas tree. (iii) Self-folding robot. Reproduced with permission from [169]. Copyright 2020 Association for Computing Machinery. (B) The conductive element was thermally actuated to change its shape and lighted up an LED. Reproduced with permission from [170]. Copyright 2016 Springer Nature. (C) The temperature sensor recovered its shape under the action of temperature and lighted up the LED. Reproduced with permission from [80]. Copyright 2016 Wiley-VCH. (D) (i) The design of the electrical safety device using the 4D-printed SMPs. (ii) Demonstration of the envisioned application using an array of 4D-printed switches with different Tt. Reproduced with permission from [171]. Copyright 2022 Elsevier.

Wang et al. [169] proposed a workflow to make 3D electronics called MorphingCircuit by combining electronic functions with forms through 4D printing technology. Briefly, the workflow entails printing a flat substrate with 4D printing technology and then assembling functional electronics on the flat substrate. When triggered by external heating, the flat structure will self-morph into a preprogrammed 3D shape. In their article, they describe the design, simulation, and fabrication of the MorphingCircuit and use it to prepare capacitors, resistors, inductors, switches, interactive Christmas trees, and light-chasing robots to demonstrate the versatility of this approach (Fig. 13A). The advent of the MorphingCircuit allows designers and researchers to design or create electronics with complex geometries that were difficult or impossible to achieve in the past, advancing the integration and development of aesthetic design and electronics.

Rodriguez et al. [170] prepared a new conductive thermoset SMPC using bisphenol F diglycidyl ether (BFDGE), epoxidized soybean oil (ESBO), and carbon nanofibers (CNF) to fabricate architecture-tunable structures with excellent conductivity, stiffness, and SME. The mechanical properties, *T*_*t*_, and conductivity of the SMPC could be tuned by varying the ratio of the CNF and each component of the thermoset resin. They used the SMPC to print a conductive element that successfully lit a light-emitting diode (LED) after shape recovery under heated conditions (Fig. 13B) and found that indirect resistive heating could also activate SME, making it promising for future electronic devices.

Zarek et al. [80] designed an electrical temperature sensor. The device was composed of a 4D-printed SMP with inkjet-printed silver nanoparticle ink on the object as its electrical contacts. The temporary shape of the device was an open electrical circuit. When heated above *T*_*t*_, its shape recovered, causing the circuit to close and light an LED to serve as a temperature warning (Fig. 13C).

Chan et al. [171] synergistically combined 4D printing and a metal plating technique to create a method for fabricating highly conductive SMPCs and fabricated a multitemperature-sensing electrical safety device that rapidly responded to ambient temperature changes (Fig. 13D). The 3 parallel circuits of this electrical safety device turned on at different temperatures to light different-colored lights, thus realizing the warning effect for different temperatures. The work provides a new development direction for designing and manufacturing electronic devices.

### Soft robots

3D-printed soft robots have received considerable attention from researchers because they enable the strategic placement of functional materials in locations that traditional instruments cannot reach. However, 3D-printed soft robots do not have autonomy, which limits their development in various applications. Combining 4D printing technology with soft robots enables them to generate the required shape conversions in response to stimuli so that they can function independently in dynamic environments.

**Fig. 14.**
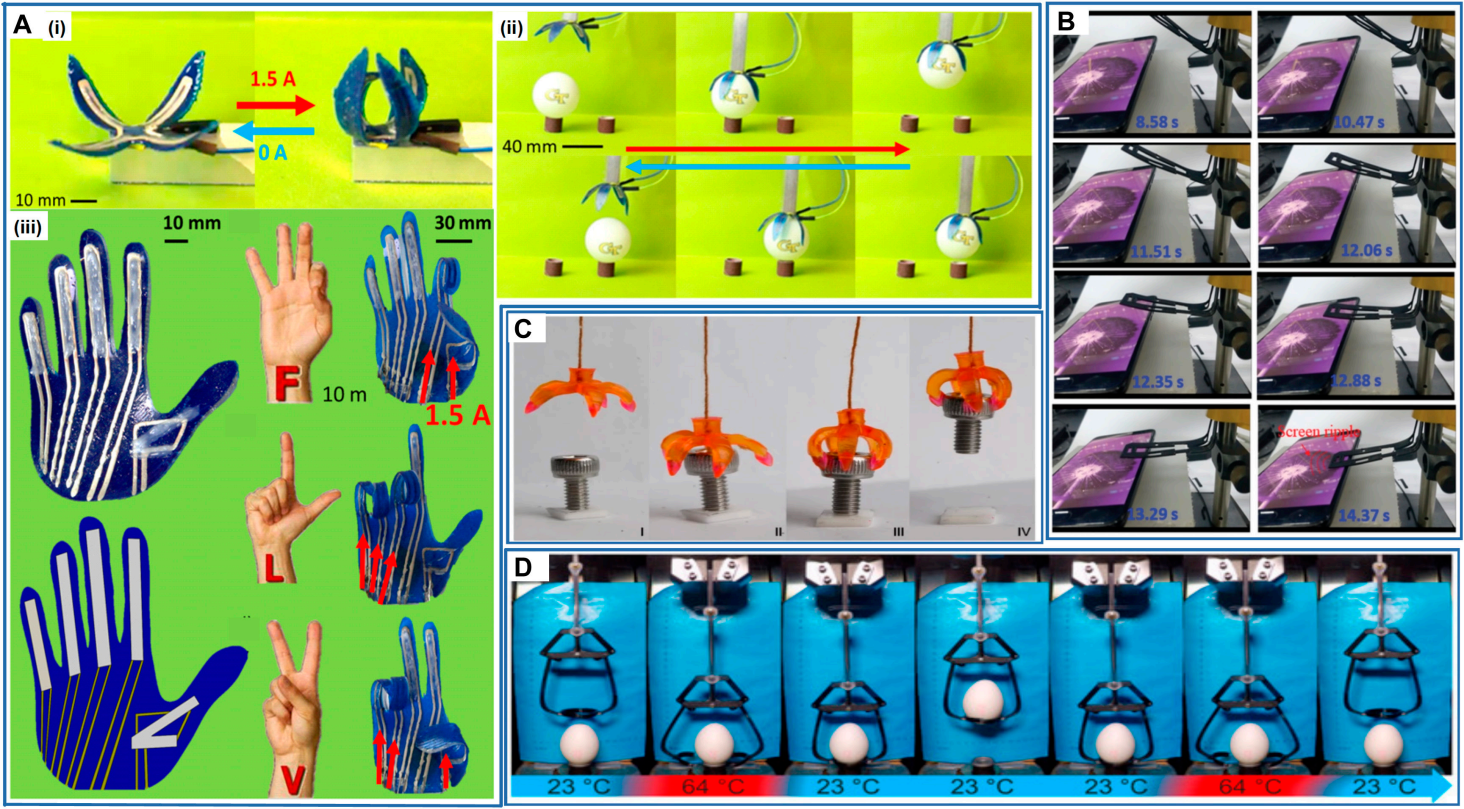
(A) (i) Four-hinge soft robotic gripper when a current of 1.5 A is applied (right) and when current is turned off (left), (ii) gripper picking and placing a ping pong ball with the current being applied in the top 3 images and turned off in the bottom 3 images, and (iii) printed hand with 5 LCE hinges for each of the 5 fingers and the formation of the letters F, L, and V by applying current to specific finger hinges to produce bending. Reproduced with permission from [172]. Copyright 2018 IOP Publishing. (B) The PISA was designed to mimic the ability of finger that could actively touch the screen. Reproduced with permission from [173]. Copyright 2020 Wiley-VCH. (C) The process of grabbing an object by a multimaterial soft robotic gripper. Reproduced with permission from [47]. Copyright 2016 Springer Nature. (D) The process of grabbing an egg by the soft robotic gripper. Reproduced with permission from [174]. Copyright 2021 Multidisciplinary Digital Publishing Institute.

Roach et al. [172] proposed a novel room-temperature printable liquid crystal elastomer (LCE) ink formulation, which enables 3D-printed LCEs to be integrated with other 3D printing methods and materials, facilitating its application to multimaterial systems. Based on this novel LCE ink formulation, they prepared a soft robotic gripper and a printed hand for sign language. They incorporated conductive wires that provide Joule heating for the electrical actuation of the soft robots. The soft robot gripper could be reversibly actuated during powering and depowering, enabling the transfer of the ping pong ball from one stand to another, and the printed hand also successfully created American sign language letters under electrical actuation, demonstrating that the 4D-printed soft robots can accurately respond to currents, even those from highly amplified neurological signals from the user (Fig. 14A).

Chen et al. [173] developed a printed integrated sensor–actuator (PISA) with bifunctional sensing and self-sensing actuation. The PISA was made of CB and PLA via an FDM printer, and they achieved the gradient bioinspired gap structure in the PISA using 4D printing technology. The PISA could actively touch the mobile phone screen while converting the mechanical signal into an electrical signal, thus gaining the ability to actively sense temperature and shape changes when it operates (Fig. 14B). This work opens new avenues for the development of multifunctional sensors, facilitating the development of future sensor–actuators and their applications in various smart scenarios.

Ge et al. [47] proposed a novel 4D printing method based on P*μ*SL to enable high-resolution multimaterial printing. They found that they could tune the material constituents and compositions to achieve highly tailored SMP thermomechanical properties. They used this 4D printing method to prepare multimaterial soft robotic grippers that could grab or release objects and demonstrated their successful screw gripping process under thermal drive (Fig. 14C).

Schönfeld et al. [174] synthesized a novel polyester urethane (PEU). They used it to prepare an actuator element in a thermo-responsive soft robotic gripper designed to precisely translate comparatively small changes into macroscopically visible and technologically relevant motions. They achieved controlled motion of the soft robotic gripper through thermally reversible shape changes of the PEU and used it to pick up transport and deposit eggs without damage (Fig. 14D). Future developments of this soft robotic gripper will expand its range of applications and enable the grasping of more challenging or larger objects.

### Other applications

As research into 4D-printed SMPs and SMPCs deepens, their application potential in various fields has been explored. In addition to the above-mentioned application directions, 4D-printed SMPs and SMPCs have also taken their unique advantages to some other fields, such as smart textiles, food, agriculture, and renewable energy. I will introduce research progress in other applications below.

**Fig. 15.**
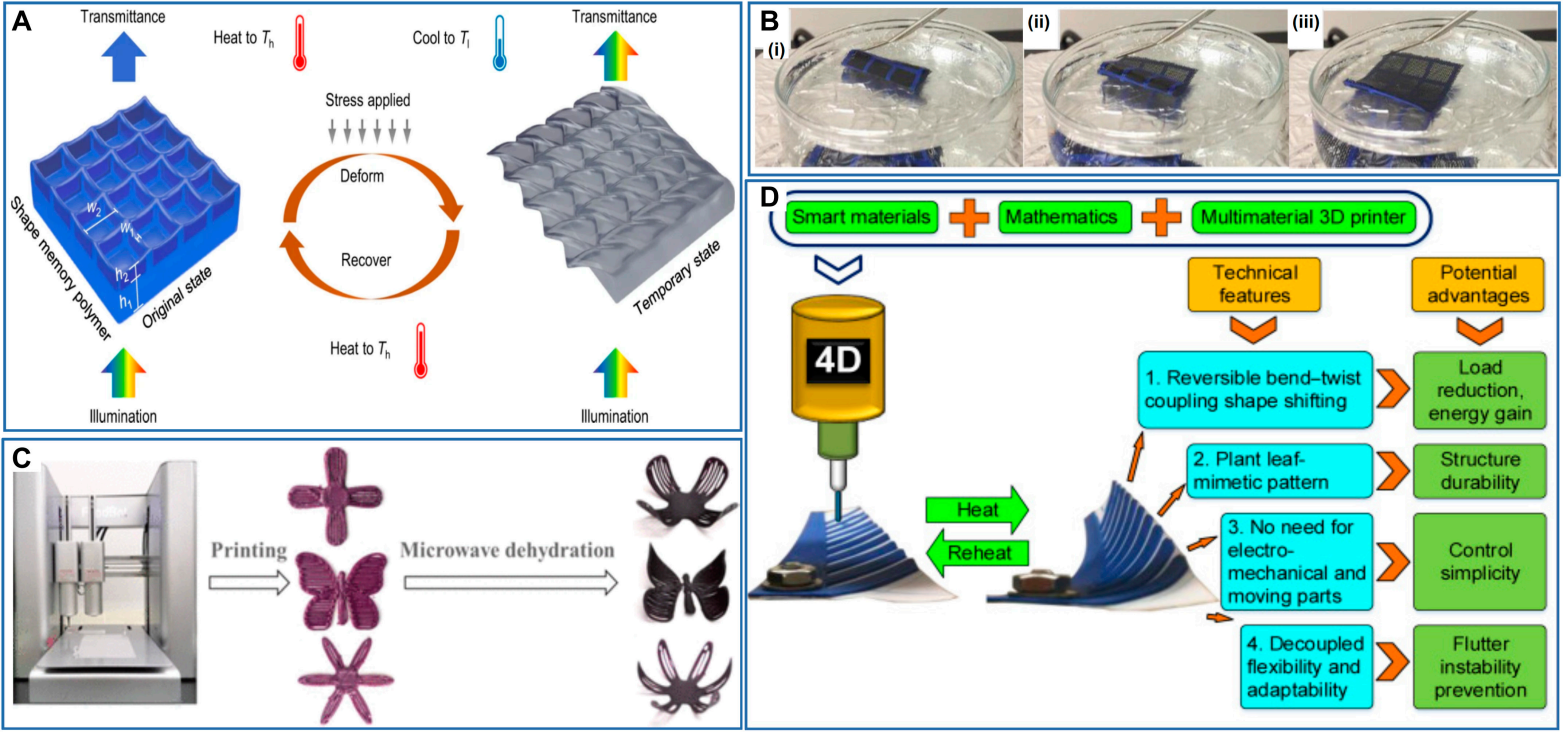
(A) Schematic of color and shape change of a constituent nanostructured element. Reproduced with permission from [48]. Copyright 2021 Springer Nature. (B) A PLA and nylon fabric combo is heated to 70 °C and rolled into a cylinder then cooled. (i to iii) The PLA nylon cylinder unfolds into its permanent flat shape when reheated in the 70 °C pool of water. Reproduced with permission from [175]. Copyright 2017 Taylor & Francis Group. (C) The process of shape changes of 4D-printed starch-based purees. Reproduced with permission from [176]. Copyright 2020 American Chemical Society. (D) The shape change and the technical advantages of the plant leaf-mimetic smart wind turbine blade. Reproduced with permission from [177]. Copyright 2019 Elsevier.

Zhang et al. [48] proposed the concept of submicron-scale 4D printing of SMPs and applied it to multicolor invisible inks by 2-photon polymer lithography (TPL) to prepare a nanostructured element. The nanostructured element can be readily produced in different colors by varying 4D printing parameters. It is capable of changing its geometry and optical properties depending on the temperature over time, thus enabling preferential transmission of certain wavelength ranges of incident white light illumination (Fig. 15A). The nanostructured elements could be used as components of next-generation greenhouse coverings to resist UV photo-oxidation for greenhouse thermoregulation.

Leist et al. [175] explored the 4D printing properties of the PLA filaments commonly used in 4D printing and printed PLA in the form of a grid structure onto nylon fabric to create a smart textile with shape memory properties. They heated the smart textile to 70 °C in hot water and rolled it into a cylindrical shape, then removed it from the hot water and cooled it to room temperature, and the smart textile stayed cylindrical after cooling. However, when it was put back into the hot water, the smart textile unraveled to its original flat shape (Fig. 15B), demonstrating its excellent shape memory properties. This study allows for custom shapes and aesthetics for clothing and promotes the development of 4D printing technology in the field of smart textiles.

He et al. [176] proposed a novel approach that uses microwave dehydration to drive the deformation of 4D-printed starch-based purees from purple sweet potatoes. They first investigated the rheological and dielectric properties and water distribution behavior of the purees. They explored the effects of salt content, fructose syrup content, and microwave power on the spontaneous deformation of the 4D-printed purees. Based on these findings, they printed starch-based complex printed models such as flowers and butterflies and successfully deformed them by microwave dehydration (Fig. 15C). Their approach allows food materials to be printed first as different 2D structures according to consumer needs and then converting such structures into 3D structures by microwave dehydration, which will become an option to enhance the consumers’ experience.

Momeni et al. [177] developed plant leaf-mimetic smart wind turbine blades by 4D printing (Fig. 15D). The smart wind turbine blades mimic the veins of plant leaves with global optimal performance, which results in relatively better mechanical properties and higher fatigue life relative to conventionally produced wind turbine blades. In addition, the smart material properties eliminate the need for conventional electromechanical systems and moving parts and solve the flutter instability issue associated with conventional wind turbine blades during bending-torsional coupling. These blades are expected to be used to manufacture eco-friendly wind turbines and have a high potential for applications in renewable energy.

## Current Challenges and Development Prospects

Since 4D printing was proposed, it has attracted increasing interest in academia and various industries, thereby leading to tremendous progress in all aspects of 4D printing and exhibiting a significant impact in various fields. However, the research and application of 4D printing are still in their infancy, and many problems remain to be solved.

First, most materials are insensitive to external stimulation. In the case of biocompatible and biodegradable materials, this significantly limits the range of materials suitable for 4D printing. When such SMPs and SMPCs are used for clinical treatment, the maximum temperature cannot exceed 45 °C to avoid thermal damage. Accordingly, controlling the shape recovery process at a suitable rate within the physiological temperature range is challenging for 4D printing for clinical treatment. Although SMPs and SMPCs for noncontact operation are suitable for biomedical applications, this aspect is less studied, and many actuation methods and materials are unsuitable for manipulating cell-filled 4D-printed implants. In addition, most SMPs and SMPCs cannot undergo reversible shape changes; therefore, they need to be re-edited before each deformation, which hinders the development and application of 4D-printed SMPs and SMPCs. Therefore, it is necessary to develop new material systems and noncontact manipulation methods suitable for the biomedical field.

Second, 4D printing technology has certain limitations. To date, a limited number of 4D printing methods have been employed, and each printing method has its drawbacks. In addition, the lack of theoretical models and special software tools for 4D printing hinders its design and research. The lack of theoretical models hampers the prediction of the effects of various parameters on the shape recovery process to aid polymer design. Furthermore, most software tools are still mainly applicable to previous manufacturing methods and do not satisfy the requirements of 4D printing. Therefore, there is a need to introduce and develop powerful 4D printing software tools and explore and optimize 4D printing techniques and theoretical models to improve the efficiency and quality of 4D printing technology.

Finally, most 4D-printed SMPs and SMPCs for biomedical applications are still in the early stages of development and require extensive animal testing and long-term in vivo characterization. For example, for 4D-printed SMPs and SMPCs used as biodegradable vascular stents, further research is needed on the appropriate degradation time for the treatment of restenosis after restoring vascular patency, and more in vivo tests and optimization methods are needed before these can be widely used in the clinic to ensure that they are fully functional in clinical treatment.

With the development of 4D printing technology, these challenges will be gradually solved, and innovative, better-performing products will be produced, especially in the biomedical field. 4D-printed SMPs and SMPCs will help provide personalized and customized clinical treatment plans based on a patient’s disease condition to reduce patient pain and help speed recovery. 4D-printed SMPs and SMPCs for biomedical applications are continuously being developed, and more multifunctional smart medical instruments will be used in future biomedical purposes, surpassing the technological platform of traditional medical devices and promoting the development of minimally invasive clinical surgery, slow drug release, tissue and organ replacement.
